# Anti-Influenza Virus Activity and Chemical Components from the Parasitic Plant *Cuscuta japonica* Choisy on *Dimocarpus longans* Lour.

**DOI:** 10.3390/molecules25194427

**Published:** 2020-09-26

**Authors:** Ju-Chien Cheng, Chia-Ching Liaw, Ming-Kuem Lin, Chao-Jung Chen, Chien-Liang Chao, Chih-Hua Chao, Yueh-Hsiung Kuo, Yen-Po Chiu, Yu-Shin Peng, Hui-Chi Huang

**Affiliations:** 1 Department of Medical Laboratory Science and Biotechnology, China Medical University, Taichung 404, Taiwan; jccheng@mail.cmu.edu.tw (J.-C.C.); albert50137@gmail.com (Y.-P.C.); sean56789912@gmail.com (Y.-S.P.); 2 Division of Chinese Materia Medica Development, National Research Institute of Chinese Medicine, Taipei 112, Taiwan; liawcc@nricm.edu.tw; 3 Department of Biochemical Science and Technology, National Chiayi University, Chiayi 60004, Taiwan; 4 Department of Chinese Pharmaceutical Sciences and Chinese Medicine Resources, China Medical University, Taichung 404, Taiwan; linmk@mail.cmu.edu.tw (M.-K.L.); kuoyh@mail.cmu.edu.tw (Y.-H.K.); 5 Graduate Institute of Integrated Medicine, China Medical University, Taichung 404, Taiwan; cjchen@mail.cmu.edu.tw; 6 Proteomics Core Laboratory, Department of Medical Research, China Medical University Hospital, Taichung 404, Taiwan; 7 Sinphar Pharmaceutical Co., Ltd., Sinphar Group, Yilan 269, Taiwan; chaokmc@gmail.cmu.edu.tw; 8 School of Pharmacy, China Medical University, Taichung 404, Taiwan; chchao@mail.cmu.edu.tw; 9 Chinese Medicine Research Center, China Medical University, Taichung 404, Taiwan; 10 Department of Biotechnology, Asia University, Taichung 413, Taiwan; 11 Master Program for Food and Drug Safety, China Medical University, Taichung 404, Taiwan

**Keywords:** *Cuscuta japonica* Choisy, *Dimocarpus longans* Lour., parasitic plant, influenza A virus (IAV), cuscutasides

## Abstract

Dodder (*Cuscuta* spp.) is a parasitic weed damaging many plants and agricultural production. The native obligate parasite *Cuscuta japonica* Choisy (Japanese dodder) parasitizes *Dimocarpus longans* Lour., *Ficus septica* Burm. F., *Ficus microcarpa* L.f., *Mikania micrantha* H.B.K. and *Melia azedarach* Linn, respectively. Five Japanese dodders growing on different plants exhibit slightly different metabolites and amounts which present different pharmacological effects. Among these plants, a significant antiviral activity against influenza A virus (IAV) was found in Japanese dodder parasitizing on *D. longans* Lour. (CL). To further explore methanol extract components in Japanese dodder (CL), four undescribed aromatic glycosides, cuscutasides A–D (compounds **1**–**4**) were isolated, together with twenty-six known compounds **5**–**30**. The chemical structures of **1**–**4** were elucidated using a combination of spectroscopic techniques. The eighteen isolated compounds were evaluated for antiviral activity against IAV activity. Among them, 1-monopalmitin (**29**) displayed potent activity against influenza A virus (A/WSN/1933(H1N1)) with EC_50_ 2.28 ± 0.04 μM and without noteworthy cytotoxicity in MDCK cells. The interrupt step of **29** on the IAV life cycle was determined. These data provide invaluable information for new applications for this otherwise harmful weed.

## 1. Introduction

Influenza A virus (IAV) is a highly contagious, epidemic etiology respiratory illness that has affected 3–5 million population annually worldwide with high mortality and morbidity [1]. This virus has developed multiple strategies to evade host immune defenses including continuous genetic changes through mutation and re-assortment [2]. The influenza viral life-cycle is well known. The viral ion channel protein M2 and viral releasing neuraminidase are the clinical therapeutic targets present time. However, most currently circulating IAV’s are resistant to M2 inhibitors, neuraminidase inhibitors are the only class of recommended anti-IAV treatments to date [3]. It is still unmet for anti-IAV agents. Plants have long been thought a potential source of anti-viral drugs. With the development of new influenza strains resistant to current commercially antiviral drugs, a wider range of influenza antiviral compounds is needed [4].

*Cuscuta japonica* Choisy (Japanese dodder) is a unique kind of holoparastic vine from the Convolvulaceae family. Dodder is completely dependent on the host plant for nourishment to survive, so its phytochemical constituents depend on the type of host [5]. When *Cuscuta* sp. parasitizes a medicinal plant such as *Taxus brevifolia* and grows thereon and can produce secondary metabolites lead to a pharmaceutical component (camptothecin) [6]. Dodder is a parasitic weed and spreads rapidly, afflicting many commercial crops, ornamentals and native plants by virtually decimating or killing them [7]. Herbicides are able to control the parasitic weed and are surely phytotoxic to the host plants. There are thus advantages in utilizing this harmful weed as a source of valuable products, including increased effective management and reduced negative impacts on the environment.

*Cuscuta* is a genus of about 170 species of threadlike, yellowish, orange or reddish parasitic plants distributed mainly in North and South America. A few species are found in Asia and Europe. The *C. chinensis* Lam., *C. japonica* Choisy, and *Cuscutae* Semen seeds are a well-known traditional medicinal material, commonly used for improving sexual function, tonifying the livers and kidneys, reducing urination, treating aches and weakness in the loins and knees, and treating pharyngitis [8]. Investigation into the phytochemistry of *Cuscuta* spp. seeds has so far led to the isolation of many compounds, consisting of flavonoids, alkaloids, steroids, fatty acids, lignans [7], aromatics [9], resin glycosides [10] and polysaccharides [7]. These compounds exhibit a range of pharmacological effects such as hepatoprotective, anti-osteoporotic, immune regulation, neuroprotection, antioxidative, anti-aging, cytotoxic, renoprotective, reproductive system, prevention of abortion, antimutagenic effect, antidiabetic, cardioprotective, antidepressant, and anti-inflammatory activities [7]. However, the parasitic plant *C. japonica* growing onto a plethora of different plants has only been the subject of rare studies concerning host-parasite relationships and phytochemical constituents. In this work we analyzed the chemical fingerprint in five Japanese dodders (*C. japonica*) parasitizing on different plants using HPLC, thus evidencing slight differences.

In the course of screening for anti-virus (H1N1) substances from five Japanese dodders parasitizing different plants (*Dimocarpus longans* Lour., *Ficus septica* Burm. F., *Ficus microcarpa* L. f., *Mikania micrantha* H. B. K. and *Melia azedarach* Linn., respectively), Japanese dodder parasitizing on *D. longans* Lour. (CL) exhibited significantly suppressed anti-virus activation. Japanese dodder (CL) was not previously studied from a phytochemical viewpoint. During research efforts to discover bioactive compounds from the methanol extract of the whole plant Japanese dodder (CL), four previously undescribed aromatic glycosides **1**–**4** and four known aromatic glycosides **5**–**8**, two caffeoylquinic acid derivatives **9**–**10**, five lignans **11**–**15**, four tropane alkaloids **16**–**19**, two indole alkaloids **20**–**21**, five flavanoids **22**–**26**, two aromatic compounds **27**–**28**, and two fatty acids **29**–**30** were obtained. Eighteen of the isolated compounds were subjected to anti-IAV activity assays and the effective compound that interrupted the IAV life cycle was determined.

## 2. Results

**Figure 1:**
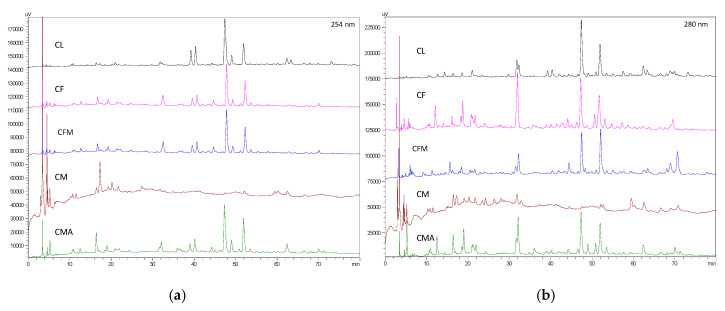
HPLC-DAD chromatogram of five Japanese dodder parasitizing on different plants, *D. longans* Lour. (CD), *F. septica* Burm. f. (CF), *F. microcarpa* L. f. (CFM), *M. micrantha* H. B. K. (CM), and *M. azedarach* Linn (CMA), respectively. Wavelengths: 254 nm (**a**) and 280 (**b**) nm.

**Figure 2:**
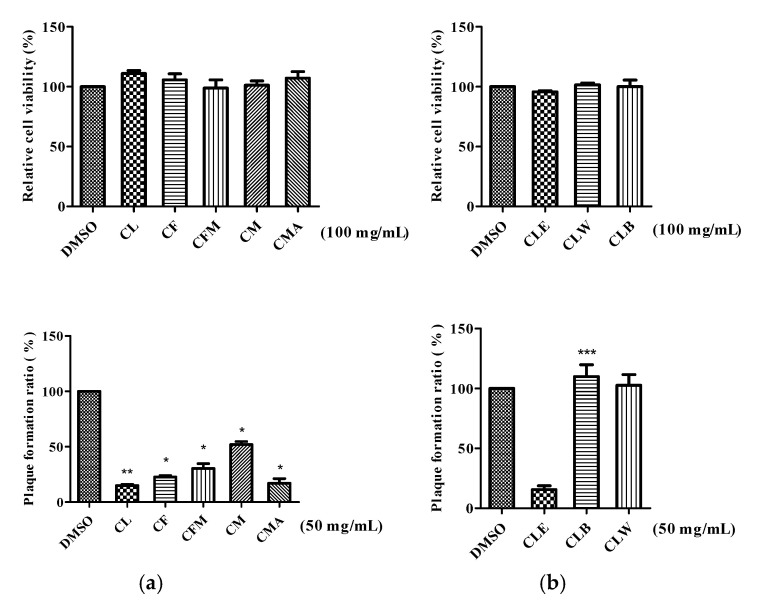
Anti-IAV activity of the extracts, fractions, and isolated compounds using the viral plaque reduction assay on MDCK cells with A/WSN/33 (H1N1) virus. (**a**) The anti-IAV activity of the CL, CF, CFM, CM, and CMA extracts. Cell viability was shown in upper panel and plaque forming reducing was shown in bottom panel. (**b**) The anti-IAV activity of the EtOAc (CLE), BuOH (CLB), and H_2_O (CLW) layers from CL extract. Cell viability was shown in the upper panel and plaque forming reducing is shown in the bottom panel. The data are presented as mean ± SD of three independent experiments and were normalized to the data of solvent control (* *p* < 0.05, ** *p* < 0.01 and *** *p* < 0.001).

**Figure 3:**
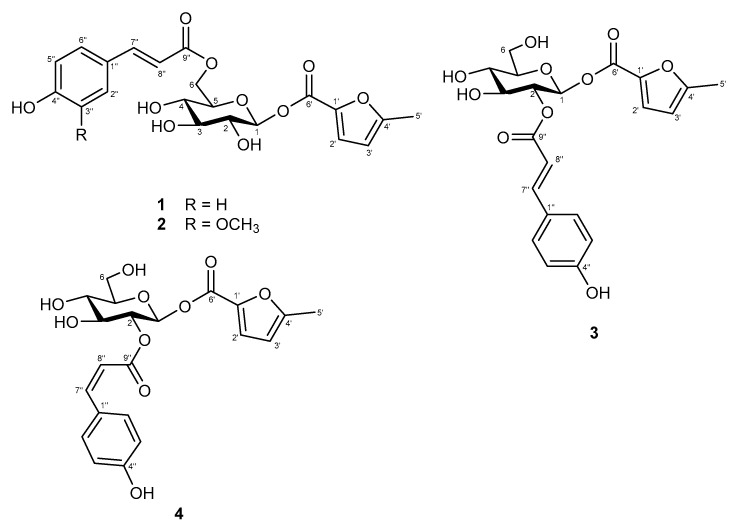
Chemical structures of compounds **1**–**4**.

The chemical constituent patterns of five Japanese dodder parasitizing on different plants, *D. longans* Lour. (CL), *F. septica* Burm. F. (CF), *F. microcarpa* L. f. (CFM), *M. micrantha* H. B. K. (CM) and *M. azedarach* Linn. (CMA), respectively, detected by HPLC-DAD analyses were slightly different (Figure 1). The MeOH extract (100 μg/mL) of Japanese dodder parasitizing on *D. longans* Lour. (CL) showed anti-IAV activity with no cytotoxicity in Madin-Darby canine kidney (MDCK) cells (Figure 2a). Among them, the most active extract from Japanese dodder (CL) was further subjected to solvent partitioning and three layers were obtained, including ethyl acetate (CLE), *n*-butanol (CLB), and water layers (CLW). The CLE further showed stronger anti-IAV activity at 50 μg/mL without cytotoxicity against MDCK cells at 100 μg/mL (Figure 2b). To understand the source of the active compound in the CLE extract, we were isolated by employing diaion HP-20, Sephardex LH-20, silica gel, and C_18_ column chromatography and further purified by RP-HPLC to obtain 30 compounds, including four new compounds **1**–**4** (Figure 3) and twenty-six known compounds.

### 2.1. Structural Elucidation of New Compounds

**Figure 4:**
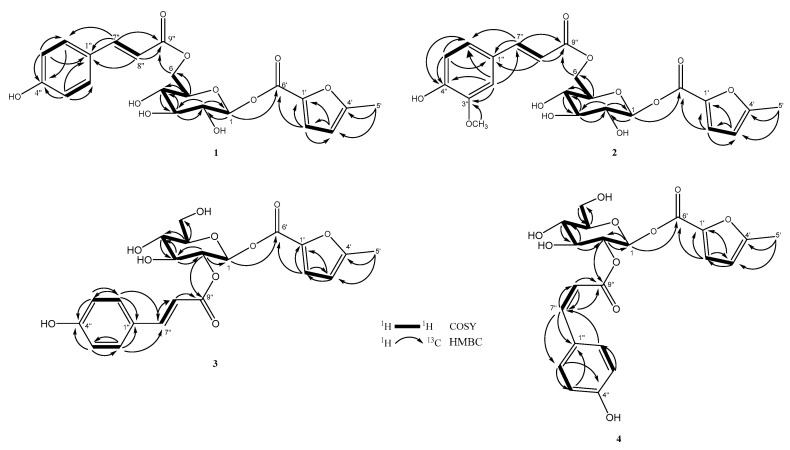
Key ^1^H-^1^H COSY and HMBC correlations of compounds **1**–**4**.

Compound **1** was isolated as a white amorphous solid. Its molecular formula was deduced to be C_21_H_22_O_10_ from the analysis of its ^13^C-NMR spectrum and the HRESIMS molecular ion peak at *m/z* 433.1123 [M − H]^−^, calculated for 433.1129), corresponding to 11 degrees of unsaturation. The UV absorption maxima at λ_max_ of 207 and 309 nm implied the presence of a conjugated chromophore, while the IR spectrum exhibited absorption bands due to a hydroxyl (3361 cm^−1^), a conjugated carbonyl (1693 cm^−1^), and an 1,4-disubtituent aromatic ring (1604, 1514 and 831 cm^−1^). The ^1^H-NMR spectrum of **1** (Table 1) displayed downfield signals of 1,4-disubstitued aromatic protons at δ_H_ 7.56 (2H, d, *J* = 8.5 Hz, H-2″/6″) and 6.79 (2H, d, *J* = 8.5 Hz, H-3″/5″), and a pair of *trans* olefinic protons at δ_H_ 7.60 (1H, d, *J* = 16.0 Hz, H-7″) and 6.37 (1H, d, *J* = 16.0 Hz, H-8″) for the *trans p*-coumaroyl moiety, along with an anomeric proton at δ_H_ 5.05 (1H, d, *J* = 7.5 Hz, H-1) which resonances with one di-oxygenated carbon at δ_C_ 100.9 for a sugar moiety. The ^13^C-NMR (Table 2) and DEPT spectra of **1** showed the obvious resonances for 10 carbons, which were resolved as four aromatic methine C-2″/C-6″ (δ_C_ 131.5 × 2) and C-3″/C-5″ (δ_C_ 116.9 × 2), two aromatic quaternary C-1″ (δ_C_ 127.3) and C-4″ (δ_C_ 161.5), two olefinic methine C-7″ (δ_C_ 147.1) and C-8″ (δ_C_ 114.9), one unsaturated carbonyl carbon C-9″ (δ_C_ 169.1), and one anomeric carbon C-1 (δ_C_ 100.9). These data suggested that **1** is a glycoside derivative with a para-coumaroyl moiety [11]. The correlation between H-1 to H-6 [δ_H_ 5.05 (H-1), 3.46 (H-2), 3.48 (H-3), 3.37 (H-4), 3.77 (H-5), 4.23 and 4.55 (H_2_-6)] showed a coupling network in the ^1^H-^1^H COSY spectrum, which proved the presence of the sugar unit to be a glucospyranoside (Figure 4). The β-configuration of glucopyranose was determined through NOESY correlations and coupling constant [12]. HMBC correlations between the H-6 and carbonyl carbon (δ_C_ 169.1, C-9″) indicated the presence of *p*-coumaroyl moiety at glucose methylene signal (δ_C_ 64.7, C-6). Apart from the above observation, further ^13^C-NMR spectrum analysis revealed the other signals for a carbonyl (δ_C_ 171.4), two quaternary carbons (δ_C_ 167.5 and 164.9), two olefinics (δ_C_ 101.6 and 92.0), and a methyl group (δ_C_ 19.8) carbons for this moiety. The additional signals were deduced from the resonances at δ_H_ 6.09 (1H, d, *J* = 1.0 Hz, H-3′), 5.72 (1H, d, *J* = 1.0 Hz, H-2′) and 2.20 (3H, s, H_3_-5′), which were supported by corresponding signals at δ_C_ 101.6 (C-3′), 92.0 (C-2′) and 19.8 (C-5′) in the HSQC spectrum. Combined with HMBC correlations, the moiety was established as 5-methyl-2-furoyl. The position of this 5-methyl-2-furoyl unit was assigned unambiguously as C-1 by HMBC correlation between the anomeric proton (δ_H_ 5.05, H-1) to the carbonyl (δ_C_ 171.4, C-6′) (Figure 4). Hence the structure of **1** was established as 6-*O*-(*E*)-*p*-coumaroyl-1-*β*-*O*-(5-methyl-2-furoyl)-β-d-glycopyranoside, and the compound was given the trivial name cuscutaside A.

Compound **2** was obtained as a white amorphous powder. The molecular formula C_22_H_24_O_11_ was determined based on the HRESIMS ion at *m*/*z* 503.0947 [M + K]^+^ (calculated for 503.0950), which showed 11 indices of unsaturation. The IR spectrum exhibited characteristic absorptions for a hydroxyl (3416 cm^−1^), a conjugated carbonyl (1692 cm^−1^), and an aromatic ring (1605 and 1514 cm^−1^). Inspection of ^1^H- and ^13^C-NMR data of compound **2** (Table 1 and Table 2) indicated similar NMR features to those of **1** except that it differed in the benzoate substitution patterns and the presence of one additional methoxy group (δ_H_ 3.92/δ_C_ 56.7). In the ^1^H-NMR data revealed the ABX protons of a 1,3,4-trisubstituted aromatic ring at δ_H_ 7.24 (1H, d, *J* = 2.0 Hz, H-2″), 7.09 (1H, dd, *J* = 8.5, 2.0 Hz, H-6″), and 6.81 (1H, d, *J* = 8.5 Hz, H-5″) which in combination with the ^13^C-NMR data suggested the appeared as a feruloyl group. The methoxy group (δ_H_ 3.92) attached at C-3‴ of feruloyl group were confirmed by its NOESY correlation with H-2‴ (δ_H_ 7.24, d, *J* = 2.0 Hz) and its HMBC correlation with C-3‴ (δ_C_ 149.6). The full chemical structure of **2** was further confirmed by ^1^H-^1^H COSY, HSQC, and HMBC correlations (Figure 4). Thus, the structure of **2** was determined as 6-*O*-feruloyl-1-*O*-(5-methyl-2-furoyl)-1-β-d-glycopyranoside and it was named cuscutaside B.

Compound **3** was obtained as a white amorphous powder and [α]${}_{D}^{25}$ + 15.3 (*c* 0.1, MeOH). The molecular formula, C_21_H_22_O_10_, was deduced from HRESIMS (*m*/*z* 457.1105 [M + Na]^+^, calculated 457.1105). The UV spectrum showed absorption maxima at *λ*_max_ of 205 and 312 nm. The IR absorptions at 1602, 1514, and 831 cm^−1^ indicated an 1,4-disubstited aromatic system. The IR and UV spectra similarity of **3** with those of **1** suggested that they have a similar *p*-coumaroyl glycoside. Detailed analysis of the ^1^H- and ^13^C-NMR spectra (Table 1 and Table 2) of **3** with those of **1** indicated that most of the resonances in **3** were the same as those of **1**. The significant difference between **1** and **3** was that the *trans*-*p*-coumaroyl moiety linked to C-6 of glucose in **1** and connected to C-2 of glucose in **3**, which could be inferred from the γ-effect. The position of the *p*-coumaroyl moiety of **3** was compared with that of **1**, which showed upfield shifts of C-1 (δ_C_ 98.9) by 2.0 ppm, of C-3 (δ_C_ 75.9) by 1.9 ppm and of C-6 (δ_C_ 62.2) due to γ-effect by 2.5 ppm and a downfield shift of C-5 (δ_C_ 78.9) by 2.5 ppm without γ-effect and of H-2 (δ_H_ 5.07) by 1.61 ppm according to –OH substituent change to ester function. This was further confirmed thought the observation of a HMBC correlation from δ_H_ 5.07 (H-2) to a carbonyl carbon at δ_C_ 168.2 (C-9″) and from δ_H_ 6.36 (H-8″) to a carbonyl carbon at δ_C_ 168.2 (C-9″), in addition to the consequence of ^1^H-^1^H COSY correlation fragment (Figure 4) of δ_H_ 5.34 (H-1)/5.07 (H-2)/3.71 (H-3)/3.51 (H-4)/3.57 (H-5)/3.74, 3.96 (H_2_-6). The chemical shift and *J* values (*J* = 8.0 Hz) of its anomeric proton indicated the β-configuration of glucose. The previously observed a further HMBC correlation of H-1 to C-6′ positioned the remaining 5-methyl-2-furoyl ester at C-1. Accordingly, the structure of **3** was determined to be 2-*O*-(*E*)-*p*-coumaryl-1-*O*-(5-methyl-2-furoyl)-1-β-d-glycopyranoside and named cuscutaside C.

Compound **4** was purified as a white amorphous powder and its molecular formula of C_21_H_22_O_10_ was determined on the basis of HRESIMS at *m*/*z* 433.1113 ([M − H]^−^, calculated 433.1129). The spectroscopic data of **4** was very similar to and shared many features with **3**. Comparison of the NMR data (Table 1 and Table 2) for **3** and **4** readily identified **4** as having the same a 5-methyl-2-furoyl unit substituted at C-1 and a *p*-coumaroyl moiety attached to C-2′ of a glycopyranosyl moiety. However, obviously different signals were noted in the ^1^H-NMR chemical shift for δ_H_ 6.90 (d, *J* = 12.5 Hz, H-7″) and δ_H_ 5.80 (d, *J* = 12.5 Hz, H-8″) of **4** compared to **3**. The geometry of double bonds was determined to be *cis* by the magnitude of the coupling constant *J*_H-7__″/8__″_ (12.5 Hz). In the HMBC spectrum, δ_H_ 5.03 (H-2), 6.90 (H-7″), and 5.80 (H-8″) exhibited interactions with δ_C_ 168.2 (C-9″). The relative configuration of glucosyl moiety was also determined through both NOESY correlations and coupling constants. The magnitude of the coupling constants for H-1 (*J* = 8.0 Hz) was also constructive of axial-axial couplings and established a β-d-glucose moiety. Thus, compound **4** was deduced as 2-*O*-(*Z*)-*p*-coumaryl-1-*O*-(5-methyl-2-furoyl)-1-β-d-glycopyranoside and named cuscutaside D. 

**Figure 5:**
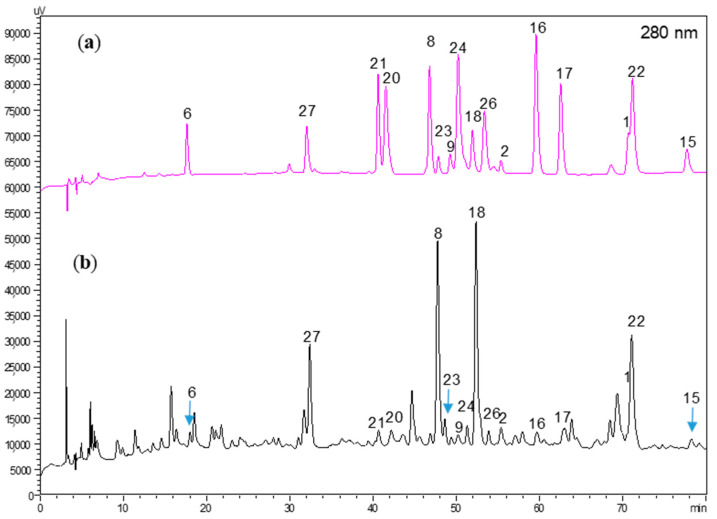
HPLC chromatograms of (**a**) the 16 marker compounds and (**b**) the CLE layer at optimum detection wavelength (280 nm).

Twenty-six known metabolites were isolated and identified as four aromatic glycosides, 6-*O*-*p*-coumaroyl-glucopyranose (**5**), 6′-*O*-cinnamoyl-glucopyranose (**6**) [13], 6′-*O*-(*E*)-*p*-coumaroyl-2-*O*-β-d-glucopyranosyl-α-d-glucopyranoside (**7**) [14], and 4-(*E*)-*p*-coumaroyl-glucopyranose (**8**) [15], two caffeoylquinic acid derivatives, 3,5-dicaffeoylquinic acid (**9**) [16], and methyl 3,5-dicaffeoylquinate (**10**), five lignans, clemaphenol A (**11**) [17], (+)-pinoresinol (**12**), (+)-syringaresinol (**13**) [18], bombasinol A (**14**) [19], and (+)-epipinoresinol (**15**) [20], four tropane alkaloids, *p*-coumaroyltyramine (**16**) [21], *N*-*trans*-feruloytyramine (**17**) [22], *N*-(*p*-*cis*-coumaroyl)tyramine (**18**) [23], and *N*-*cis*-feruloyltyramine (**19**) [24], two indole alkaloids, indole-3-carboxaldehyde (**20**) [25], and indole-3-carboxylic acid (**21**) [26], five flavonoids, querectin (**22**) [27], querectin-3-*O*-glucopyranoside (**23**) [28], quercetin-3-*O*-β-d-glucopyranosyl-(1→4)-β-d-glucopyranoside (**24**), 4′-*O*-methylquercetin-3-*O*-β-d-glucopyranoside (**25**) [29], and 3,5,7,3′-tetrahydroxy-4′-methoxyisoflavanone (**26**) [30], two aromatic compounds, *trans*-4-hydroxycinnamic acid (**27**) [31], and methyl 3,4-dihydroxycinnamate (**28**) [32], two fatty acids, 1-monopalmitin (**29**) and 1-α-linolenoylglycerol (**30**) [33] by comparing their physical and spectroscopic data with those reported in the literature. These compounds were obtained from this plant for the first time. The phytochemical composition of the 16 marker compounds from the whole plant Japanese dodder parasitizing on *D. longans* Lour. (CL) was investigated by HPLC (Figure 5).

### 2.2. Antiviral Activity against Influenza A Virus (IVA)

**Figure 6:**
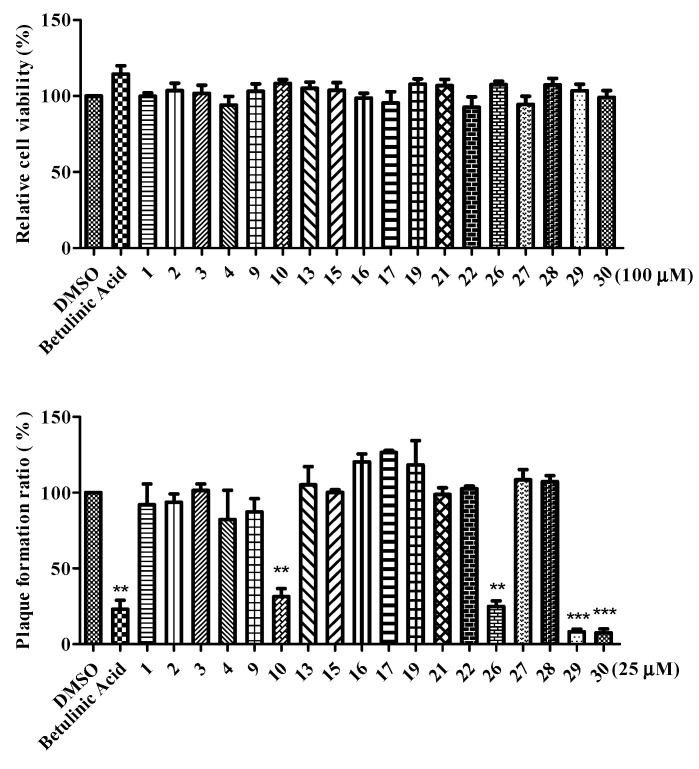
Anti-IAV activity of isolated compounds from the CLE layer. Cell viability is shown in the upper panel and plaque forming reducing shown in the bottom panel. DMSO was used as a solvent control and betulinic acid was used as a positive control. The data are presented as mean ± SD of three independent experiments and were normalized to the data of solvent control (** *p* < 0.01, and *** *p* < 0.001).

**Figure 7:**
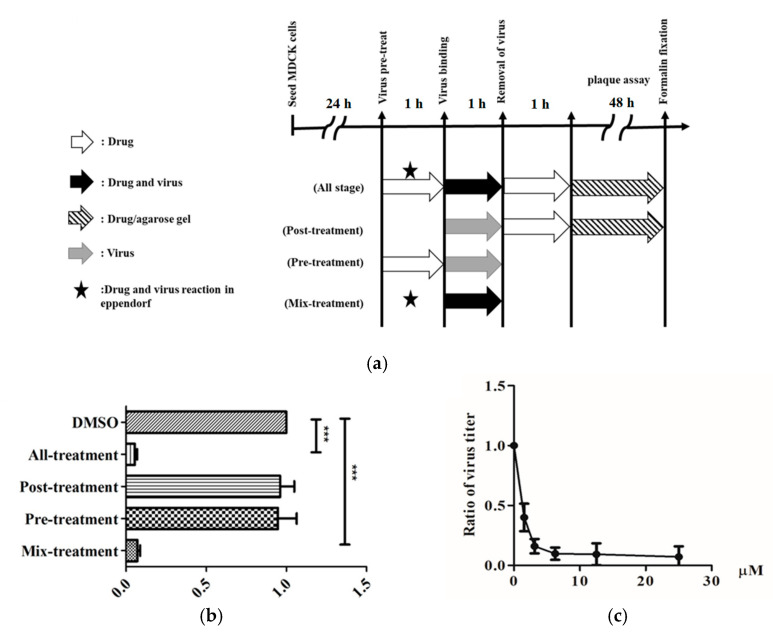
Anti-IAV assay of 1-monopalmitin (**29**). (**a**) Schematic representation of the strategies used to test the anti-IAV activity. (**b**) MDCK cells were infected with IAV (A/WSN/1933(H1N1)) and treated with **29** at the concentration of EC_90_ at the indicated time according to the different strategy presented above. (**c**) Anti-IAV activities of different concentration of **29**. DMSO was used as a solvent control. The data are presented as mean ± SD of three independent experiments and were normalized to the solvent control data (*** *p* < 0.001).

Some of the isolated compounds (**1**–**4**, **9**, **10**, **13**, **15**–**17**, **19**, **21, 22,** and **26**–**30**) were further evaluated for their antiviral activity against IAV (A/WSN/1933(H1N1)) (Figure 6). The results indicated that fatty acids **29** and **30** possessed high antiviral activities against IAV. The most effective compound **29** were further evaluated the different stages of viral infection by plaque reduction assay (Figure 7a). Compound **29** was found to effectively inhibit the entry step of IAV into cells (Figure 7b). The values of EC_50_ is 2.28 ± 0.04 μM and EC_90_ value is 4.77 ± 0.17 μM (Figure 7c).

## 3. Discussion

Comparative profiling of metabolites by HPLC-DAD in five Japanese dodders after attachment to different hosts revealed several metabolic changes and amounts. Japanese dodders parasitizing on different plants exhibited distinct anti-virus activation depending on the host. The investigation of Japanese dodder parasitizing on *D. longans* Lour. (CL) and its EtOAc fractions (CLE) showed it can effectively inhibits IAV. In this work, four new compounds **1**–**4** and twenty-six known compounds **5**–**30** were isolated from Japanese dodder (CL). It is interesting to note that 1-monopalmitin (**29**), a simple glycerol esterified fatty acid which has a role as a plant metabolite, was found to be one of anti-IAV (A/WSN/1933(H1N1)) substances from the whole plant Japanese dodder (CL). Moreover, the two fatty acids 1-monopalmitin (**29**) and 1-α-linolenoylglycerol (**30**) were more potent than betulinic acid which was used as a positive control. The fully saturated (C19:0) fatty acid, 1-monopalmitin (**29**) showed higher antiviral activities against IAV than the unsaturated (C21:3) fatty acid **30**. The current results suggest that fully saturated fatty acids play a crucial role in the anti-influenza virus activity that was observed.

## 4. Materials and Methods

### 4.1. General

1D- and 2D-NMR spectra were recorded using a Bruker DRX-500 spectrometer (Bruker Instruments, Karlsruhe, Germany) with CD_3_OD as the solvent. Infrared (IR) spectra, optical rotations, and UV spectra were measured using a JASCO FT/IR-480 plus spectrometer, a JASCO P-1020 polarimeter and a JASCO V-550 UV/Vis spectrometer, respectively (JASCO International Co. Ltd., Tokyo, Japan). HRESIMS data were obtained using a maXis impact Q-TOF mass spectrometer (Bruker Daltonik, Bremen, Germany). Sephadex LH-20 (GE healthcare, Chicago, IL, USA), Diaion HP-20 (Mitsubishi Chemical Corporation, Kyoto, Japan) and silica gel (Merck 70–230 mesh and 230–400 mesh) (Merck & Co. Inc. Darmstadt, Germany) were used for the column chromatography. The analysis HPLC was performed on a Shimadzu LC-20AT series apparatus (Shimadzu Co., Kyoto, Japan) with a prominence SPD-M20A diode array detector, a SIL-20A prominence Auto sampler, and a CTO-20A prominence column oven, using a reverse phase column (Cosmosil 5C18-AR-II column, 5 μm particle size, 250 mm × 4.6 mm i.d.) (Merck & Co., Inc., NJ, USA). Preparative HPLC was performed using a reverse phase column (Cosmosil 5C18-AR-II column, 5 μm particle size, 250 mm × 10 mm i.d.) (Merck & Co., Inc., NJ, USA) using a Shimadzu LC-6AD series apparatus with a RID-10A Refractive Index detector (Shimadzu Co.). MPLC was performed using a reverse phase column (Buchi MPLC glass column, C18, 460 mm × 36 mm i.d.) on a Buchi pump module C-601 series apparatus (Buchi Ltd., Flawi, Switzerland) without a detector. The 1D and 2D NMR spectral data of compounds **1**–**4** can be found at Supplementary Materials.

### 4.2. Plant Material

Whole *C. japonica* on *D. longans* Lour. plants used in this experiment were collected on the mountains of Nantou County, Taiwan, in August 2012. The parasitic plant was identified by Dr. Shy-Yuan Hwang, Endemic Species Research Institute. A voucher specimen of *C. japonica* on *D. longans* Lour. (No. CMR201208CL) was deposited in the Department of Chinese Pharmaceutical Sciences and Chinese Medicine Resources, China Medical University, Taichung, Taiwan. 

### 4.3. Extraction and Isolation

Whole *C. japonica* (10 kg) plants were sectioned and extracted three times with MeOH (20 L) for 72 h each time. The MeOH extract was continuously dried under reduced pressure at 45 °C to yield a brown syrup (ca. 988 g). The combined extracts were suspended on H_2_O (2 L) and then partitioned with EtOAc (2 L) and *n*-BuOH (2 L), successively. The EtOAc layer (ca. 600 g) was subjected to a silica gel CC (15 × 50 cm i.d., Merck 70–230 mesh), eluting with *n*-hexane/EtOAc (40:1, 20:1, 10:1, 5:1, 1:1, 0:1, *v*/*v*) and EtOAc/MeOH (10:1, 5:1, 4:1, *v*/*v*) in a gradient to give ten fractions (fr. 1~11). Fraction 5 (20.1 g) was subjected to Sephadex LH-20 column (3 × 90 cm, i.d.) chromatography eluting with CH_2_Cl_2_/MeOH to yield 9 subfractions (fr. 5.1–5.9). Fr. 5.3 (453.5 mg) was subjected to reverse phase (RP) MPLC (C_18_ gel, 3 × 60 cm i.d., flow rate: 20 min/mL) with gradient MeOH/H_2_O (40/60 to 100/0, *v*/*v*) to yield 8 subfractions (fr. 5.3.1~5.3.8). Fr. 5.3.1 (165.5 mg) was chromatographed using preparative RP-HPLC (Cosmosil 5C_18_-AR-II column, flow rate: 3.0 min/mL) with 38% MeOH in H_2_O to yield **13** (8.5 mg, t_R_ = 46.3 min), **14** (2.3 mg, t_R_ = 53.1 min), and **18** (4.4 mg, t_R_ = 23.8 min). Using preparative RP-HPLC (Cosmosil 5C_18_-AR-II column, flow rate: 3.0 min/mL) with 43% MeOH, **15** (8.8 mg, t_R_ = 44.8 min) were obtained from fr. 5.3.3 (32.4 mg). The subfraction 5.3.6 was subjected to RP-HPLC with 45% MeOH to obtain **29** (42.9 mg, t_R_ = 24.1 min), and **30** (59.1 mg, t_R_ = 25.6 min). Fraction 5.4 (1.98 g) was subjected to RP-MPLC (C_18_ gel, 3 × 60 cm, flow rate: 20 min/mL) with gradient MeOH/H_2_O (30:70 to 100:0, *v*/*v*) to yield 10 subfractions (fr. 5.4.1~5.4.10). Fr. 5.4.2 (293.1 mg) was subjected to preparative RP-HPLC (flow rate: 3.0 min/mL), eluting with 60% MeOH to yield 7 fractions (fr. 5.4.2.1~5.4.2.7). Fr. 5.4.2.4 (40.9 mg) was chromatographed using RP-HPLC with 43% MeOH to yield **27** (16.2 mg, t_R_ = 13.8 min). **12** (5.2 mg, t_R_ = 32.5 min) was isolated from fr. 5.4.2.6 (40.5 mg) by RP-HPLC (flow rate: 3.0 min/mL), eluting with 43% MeOH. Fr. 5.4.3 (41.7 mg) was subjected to Sephadex LH-20 column (3 × 90 cm, i.d.) chromatography eluting with MeOH to yield 6 subfractions (fr. 5.4.3.1~5.4.3.6). Fr. 5.4.3.4 (141.7 mg) was purified by RP-HPLC (45% MeOH) to yield compounds **26** (24.4 mg, t_R_ = 17.8 min). **16** (30.0 mg, t_R_ = 27.8 min) was separated by RP-HPLC with 45% MeOH from fr. 5.4.5 (149.3 mg). Fr. 5.4.6 (293.8 mg) was subjected to preparative RP-HPLC, eluting with 43%MeOH to yield **21** (4.8 mg, t_R_ = 12.0 min), **20** (4.9 mg, t_R_ = 17.0 min), **19** (7.3 mg, t_R_ = 17.8 min), **28** (61.9 mg, t_R_ = 22.1 min), **17** (55.7 mg, t_R_ = 28.3 min), and **11** (75.5 mg, t_R_ = 31.5 min). Using Sephadex LH-20 column (1.5 × 100 cm, i.d.) with isocratic MeOH, **22** (102.1 mg) and **23** (6.9 mg) were obtained from fr. 5.8 (1.67 g). Fr. 8 (14.72 g) was subjected to Sephadex LH-20 column (1.5 × 100 cm, i.d.) chromatography eluting with MeOH to yield 12 subfractions (fr. 8.1~8.12). Fraction 8.7 (2.76 g) was subjected to RP-MPLC (C_18_ gel, 3 × 60 cm, flow rate: 20 min/mL) with gradient MeOH/H_2_O (40:60 to 100:0, *v*/*v*) to yield 8 subfractions (fr. 8.7.1~8.7.8). Using preparative RP-HPLC with 50% MeOH, **1** (78.3 mg, t_R_ = 44.8 min), **2** (6.3 mg, t_R_ = 2.3 min), **3** (26.1 mg, t_R_ = 40.8 min), and **4** (4.9 mg, t_R_ = 39.1 min) were obtained from fr. 8.7.1 (650.5 mg). Subfraction 8.7.2 was further purified by RP-HPLC with 45% MeOH to give **5** (2.5 mg, t_R_ = 17.8 min), **6** (2.0 mg, t_R_ = 21.5 min), and **7** (3.4 mg, t_R_ = 18.2 min). Fr. 8.11 (60.8 mg) was further separated by RP-HPLC with 50% MeOH to yield **8** (181.8 mg, t_R_ = 7.5 min), **9** (7.7 mg, t_R_ = 10.9 min), **10** (17.8 mg, t_R_ = 16.5 min), **24** (4.1 mg, t_R_ = 14.3 min) and **25** (9.1 mg, t_R_ = 9.6 min). 

### 4.4. Spectroscopic Data

*Cuscutaside A* (**1**). White amorphous powder. [α]${}_{D}^{25}$ −86.0 (*c* 0.1, MeOH); UV (MeOH) λ_max_ (log *ε*) 207 (4.0), 227 (3.8), 309 (4.1) nm; IR (KBr) ν_max_ 3361, 1693, 1604, 1564, 1514, 1448, 1246, 1170, 1074, 1018, 831, 520 cm^−1^; ^1^H- and ^13^C-NMR data (CD_3_OD) see Table 1 and Table 2; HRESIMS *m*/*z* 433.1123 [M − H]^−^ (calculated for C_21_H_21_O_10_, 433.1129).

*Cuscutaside B* (**2**). White amorphous powder. [α]${}_{D}^{25}$ −21.0 (*c* 0.1, MeOH); UV (MeOH) λ_max_ (log ε) 207 (4.0), 308 (4.1) nm; IR (KBr) ν_max_ 3416, 1692, 1632, 1605, 1565, 1514, 1451, 1246, 1180, 1075, 822 cm^−1^; ^1^H- and ^13^C-NMR data (CD_3_OD) see Table 1 and Table 2; HRESIMS *m*/*z* 503.0947 [M + K]^+^ (calculated for C_22_H_24_O_11_K, 503.0950).

*Cuscutaside C* (**3**). White amorphous powder. [α]${}_{D}^{25}$ +15.3 (*c* 0.1, MeOH); UV (MeOH) λ_max_ (log ε) 205 (4.0), 228 (3.7), 312 (4.0) nm; IR (KBr) ν_max_ 3352, 1695, 1631, 1602, 1566, 1514, 1450, 1246, 1166, 1083, 1024, 831, 516 cm^−1^; ^1^H- and ^13^C-NMR data (CD_3_OD) see Table 1 and Table 2; HRESIMS *m*/*z* 457.1105 [M + Na]^+^ (calculated for C_21_H_22_NaO_10_, 457.1105).

*Cuscutaside D* (**4**). White amorphous powder. [α]${}_{D}^{25}$ −19.6 (*c* 0.1, MeOH); UV (MeOH) λ_max_ (log ε) 203 (3.9), 299 (3.2), 311 (3.2) nm; IR (KBr) ν_max_ 3375, 1695, 1633, 1602, 1566, 1514, 1450, 1246, 1166, 1083, 1024, 831, 526 cm^−1^; ^1^H- and ^13^C-NMR data (CD_3_OD) see Table 1 and Table 2; HRESIMS *m*/*z* 433.1113 [M − H]^−^ (calculated for C_21_H_21_O_10_, 433.1129).

### 4.5. HPLC-DAD Analysis

Five methanol crude extracts, EtOAc layer (CLE) and isolated compounds were diluted in MeOH (conc. 1.0 mg/mL), respectively. All samples were filtered through a filter cartridge (pore size of 0.22 μm) prior to analyses. The HPLC profiles of crude extracts, CLE fraction and the isolated compounds were performed on a RP-18 column at a flow rate of 1.0 mL/min and detected at 254 and 280 nm. The injection volume was 5 μL. The separation was performed using a mixture of 0.1% trifluoroacetic acid (TFA) water solution (A) and methanol (B) with the gradient elution: 0–120 min from 5% B to 55% B.

### 4.6. Anti-Inflluenza Virus Assay

The anti-IAV activity was displayed using plaque reduction assay as described previously [34]. In brief, the five crude extracts (CL, CF, CFM, CM and CMA), three fractions (CLE, CLW, and CLW), compounds (**1**–**4**, **9**, **10**, **13**, **15**–**17**, **19**, **22-24**, and **26**–**30**) cytotoxicity were determined by MTS cell viability assay at the concentration of 100 µM of each samples in MDCK cells in the first. MDCK cells were cultured in DMEM, supplemented with 10% fetal bovine serum (Gibco, Carlsbad, CA, USA) and 1% penicillin and streptomycin (PS; HyClone, Marlborough, MA, USA) at 37 °C with 5% CO_2_ incubation. The non-cytotoxic samples were selected to the following plaque reduction assay. MDCK cells (2 × 10^6^ cells/well) were pre-seeded in a six-well plate at 37 °C overnight. The cell monolayer cells was then infected with influenza virus (A/WSN/1933 (H1N1), 100 PFU/well) with 25 µM of each samples and the plaque forming assay was performed. The anti-IAV activity of each sample was calculated using the plaque number compared with that of the virus infected control. The most effective compound 29, was used to further perform the dose dependent plaque reduction assay. The 50% and 90% effective concentrations (EC_50_ and EC_90_) were defined as the concentration required for 50% and 90% inhibition of virus infection, respectively.

### 4.7. Time-Of-Addition Assay

To determine the stage of at which 1-monopalmitin (**29**) inhibits IAV infection, three time points, including post-treatment, pre-treatment and mix-treatment, were used as previous study [34]. In brief, for the stage of post treatment, the viruses infected the cells for 1 h and the cells were overlaid with 0.3% agarose gel containing the compound for 48 h. The effect of 1-monopalmitin inhibits IAV at post-infection stage was determined using the virus plaque reduction assay. For the stage of Pre-treatment, the cells were pretreated with 1-monopalmitin for 1 h followed by virus infection of the pre-treated cells for another 1 h. After the virus removed, plaque reduction assay were performed. For the stage of Mix-treatment, the viruses were pretreated with 1-monopalmitin for 1 h, and then the mixture of compound and viruses was added to the cells for another 1 h. After the mixture remove, plaque reduction assay were performed.

## 5. Conclusions

Bioactivity-guided isolation of Japanese dodder (CL) let to the isolation and identification of four new compounds **1**–**4** and twenty-six known compounds **5**–**30**. The structures were established via extensive spectroscopic investigations, including 1D and 2D NMR, UV, IR and HRESIMS techniques. The eighteen isolated compounds were evaluated for antiviral activity against IAV activity. 1-Monopalmitin (**29**) exhibits potential to be developed as a promising agent against influenza virus infection. This study is important as it explain the chemical and biological diversity of the whole plant Japanese dodder parasitizing on *D. longans* Lour. Furthermore, the results provide invaluable information for the new application of the harmful weed. 

## Figures and Tables

**Table 1:** ^1^H-NMR (500 MHz) spectroscopic data for **1**–**4** in CD_3_OD.

Position	1	2	3	4
	δ_H_, *J* in Hz			
1	5.05 (d, 7.5)	5.05 (d, 7.5)	5.34 (d, 8.0)	5.25 (d, 8.0)
2	3.46 (dd, 9.0, 7.5)	3.48 (dd, 8.5, 7.5)	5.07 (dd, 9.0, 8.0)	5.03 (dd, 9.0, 8.0)
3	3.48 (t, 9.0)	3.50 (t, 8.5)	3.71 (t, 9.0)	3.64 (t, 9.0)
4	3.37 (t, 9.0)	3.39 (t, 8.5)	3.51 (br d, 9.0)	3.47 (t, 9.0)
5	3.77 (ddd, 9.0, 7.5, 2.0)	3.78 (ddd, 8.5, 7.5, 2.0)	3.57 (m) ^a^	3.53 (m) ^a^
6	4.23 (dd, 12.0, 7.5) 4.55 (dd, 12.0, 2.0)	4.25 (dd, 12.0, 7.5) 4.57 (dd, 12.0, 2.0)	3.74 (dd, 12.0, 5.5) 3.96 (dd, 12.0, 2.0)	3.71 (dd, 12.0, 5.0) 3.90 (br d, 12.0)
2′	5.72 (d, 1.0)	5.74 (d, 2.0)	5.68 (d, 2.0)	5.66 (br s)
3′	6.09 (d, 1.0)	6.08 (d, 2.0)	5.98 (br s)	5.95 (br s)
4′	-	-	-	-
5′	2.20 (s)	2.15 (s)	2.16 (s)	2.18 (s)
2″	7.56 (d, 8.5)	7.24 (d, 2.0)	7.43 (d, 8.5)	7.63 (d, 8.5)
3″	6.79 (d, 8.5)	-	6.79 (d, 8.5)	6.73 (d, 8.5)
4″	-	-	-	
5″	6.79 (d, 8.5)	6.81 (d, 8.5)	6.79 (d, 8.5)	6.73 (d, 8.5)
6″	7.56 (d, 8.5)	7.09 (dd, 8.5, 2.0)	7.43 (d, 8.5)	7.73 (d, 8.5)
7″	7.60 (d, 16.0)	7.62 (d, 16.0)	7.67 (d, 16.0)	6.90 (d, 12.5)
8″	6.37 (d, 16.0)	6.42 (d, 16.0)	6.36 (d, 16.0)	5.80 (d, 12.5)
OCH_3_	-	3.92 s	-	-

^a^ Overlapped signals are reported without designating multiplicity.

**Table 2:** ^13^C-NMR (125 MHz) spectroscopic data for **1**–**4** in CD_3_OD.

Position	1	2	3	4
	δc			
1	100.9	100.8	98.9	98.9
2	74.5	74.4	74.4	74.4
3	77.8	77.8	75.9	76.0
4	71.7	71.7	71.1	71.2
5	76.4	76.1	78.9	78.9
6	64.7	64.7	62.2	62.2
1′	167.5	167.3	167.3	167.3
2′	92.0	92.1	91.9	91.9
3′	101.6	101.6	101.4	101.4
4′	164.9	164.8	165.1	165.1
5′	19.8	19.9	19.8	19.8
6′	171.4	171.3	171.0	171.5
1″	127.3	127.7	127.1	131.4
2″	131.5	111.9	131.4	133.8
3″	116.9	149.6	117.0	116.2
4″	161.5	151.0	161.5	162.3
5″	116.9	116.6	117.0	116.2
6″	131.5	124.6	131.4	133.8
7″	147.1	147.4	147.5	147.5
8″	114.9	115.1	114.7	115.9
9″	169.1	169.1	168.2	168.2
OCH_3_	-	56.7	-	-

## Supplementary Materials

The 1D and 2D NMR spectra of compounds **1**–**4** are available online.
