# Supplementary material for: Anti-Influenza Virus Activity and Chemical Components from the Parasitic Plant Cuscuta japonica Choisy on Dimocarpus longans Lour
Source: Molecules. 2020 Sep 26;25(19):4427. doi: 10.3390/molecules25194427 (PMC7582473; doi:10.3390/molecules25194427)
Supplement: Supplementary file 1 [file molecules-25-04427-s001.pdf]

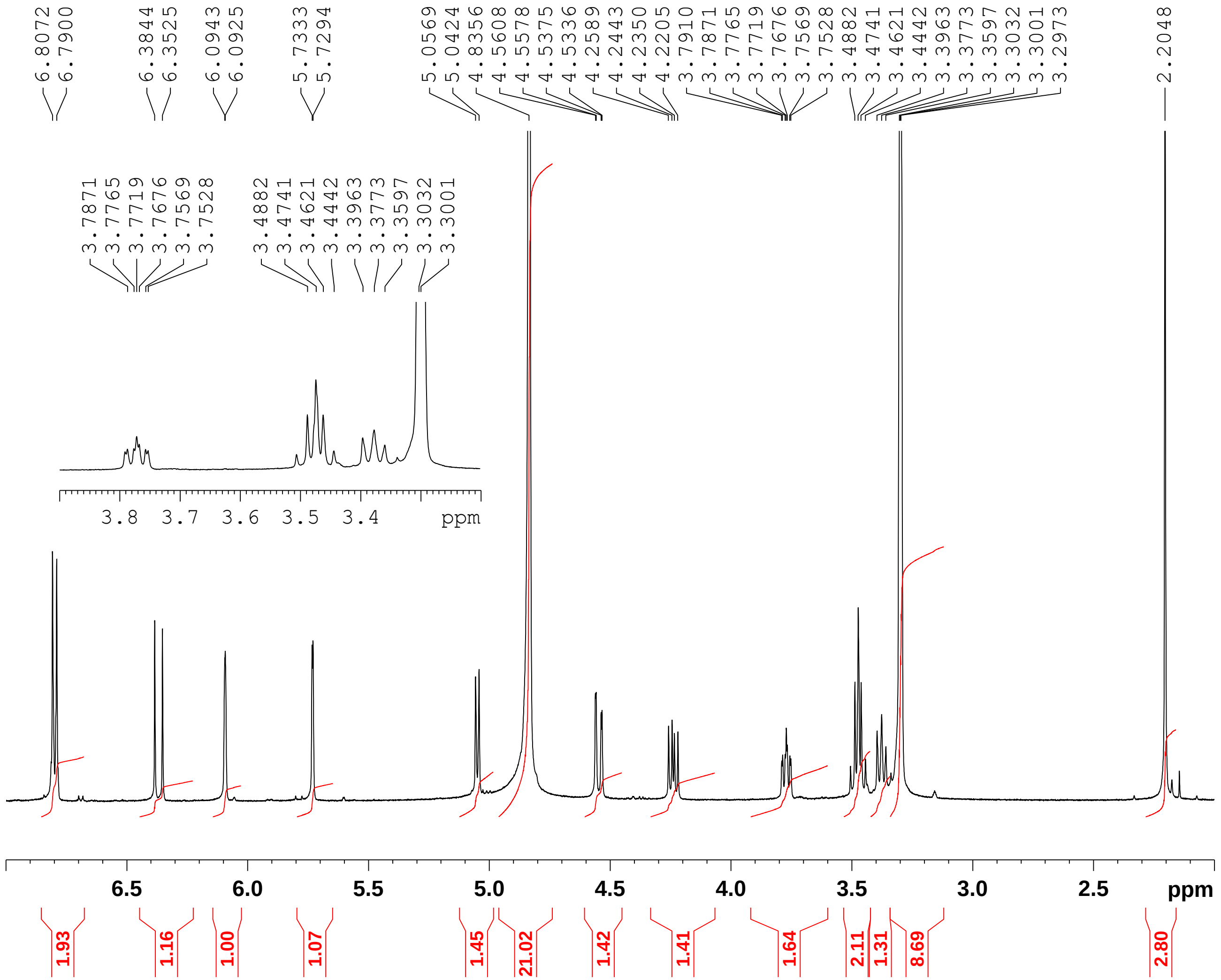

Figure 1-1S. ^{1}H NMR spectrum of compound **1** (CD_3OD, 500 MHz).

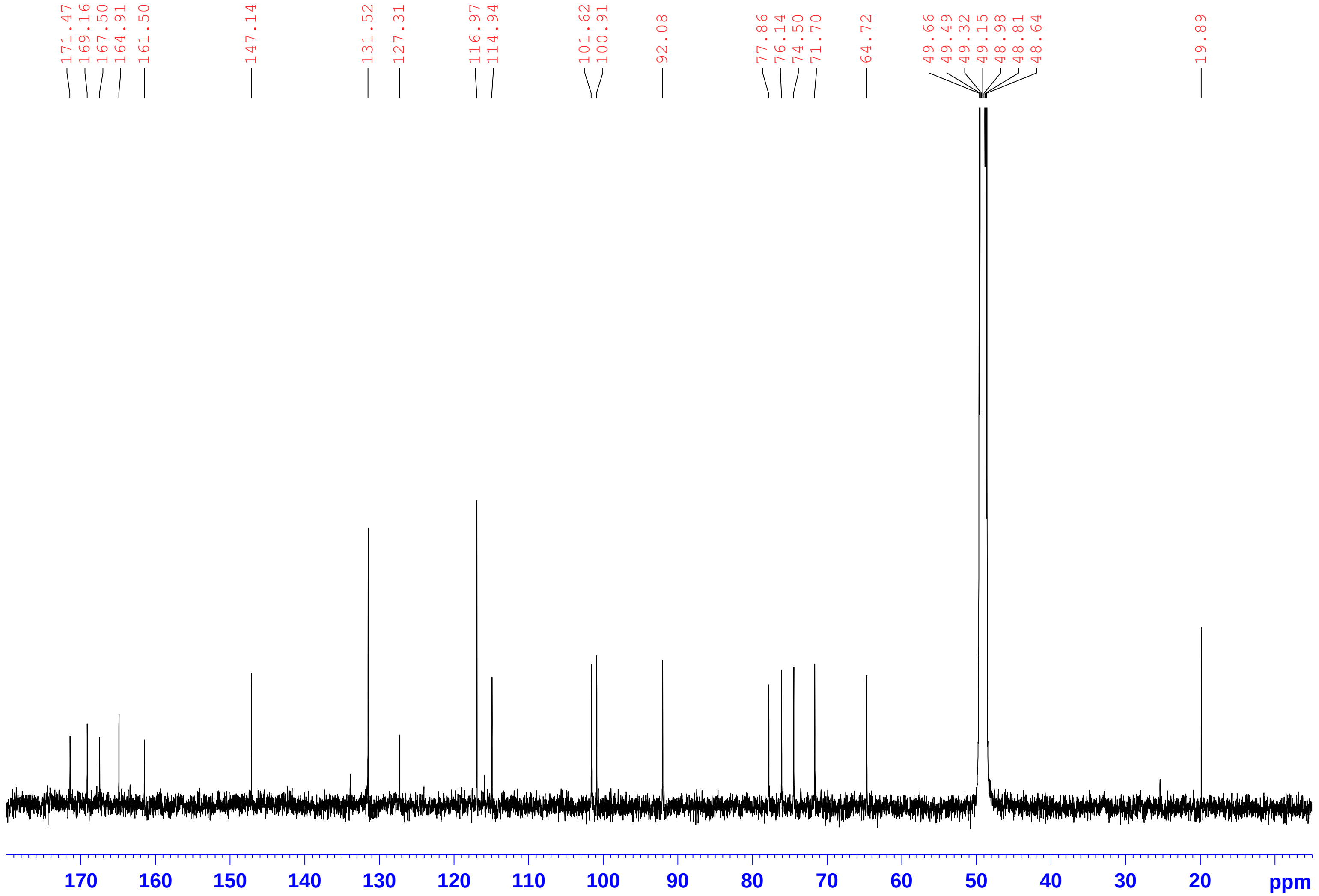

Figure 1-2S. ^{13}C NMR spectrum of compound **1** (CD_3OD, 125 MHz).

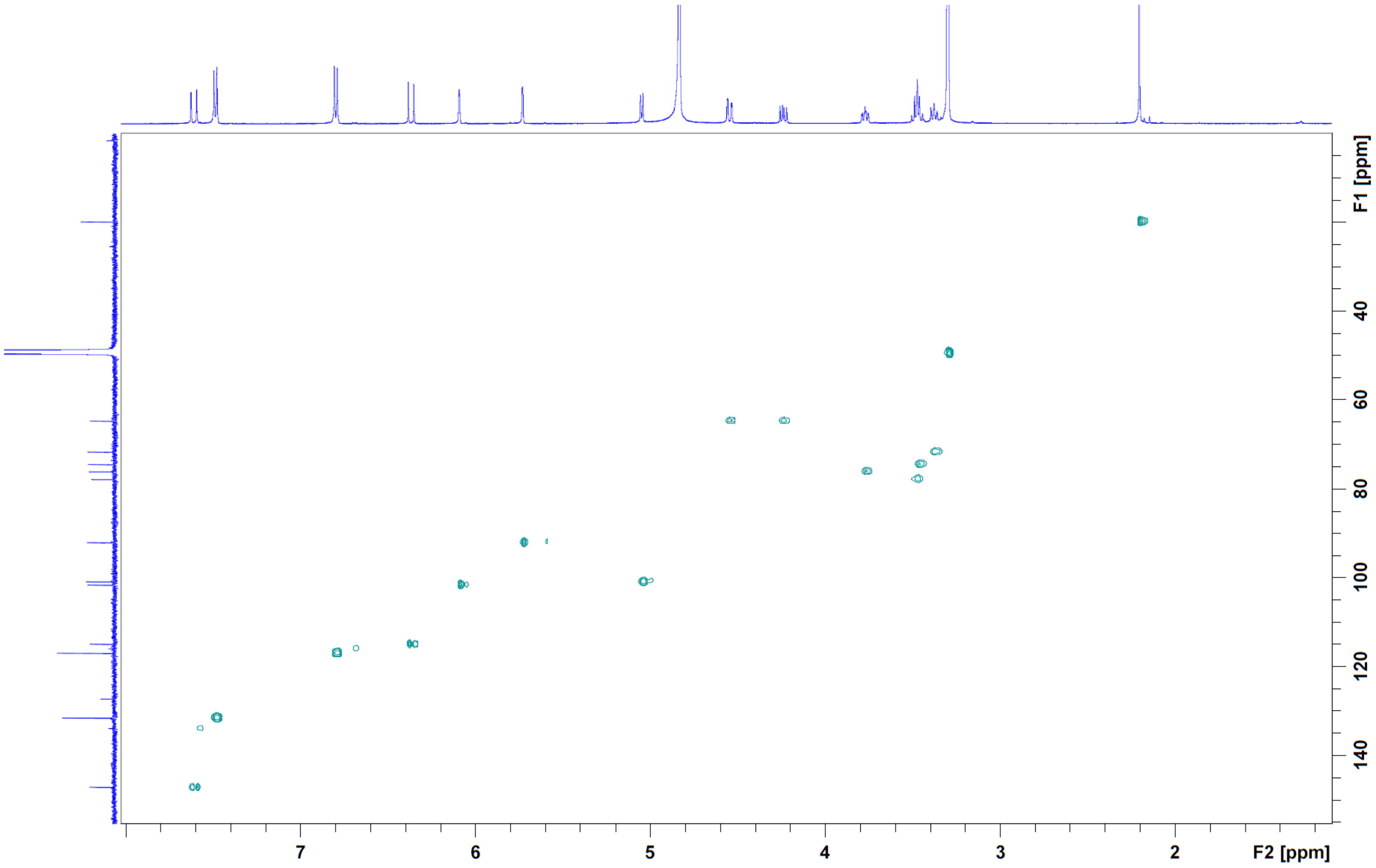

Figure 1-3S. HSQC spectrum of compound **1**.

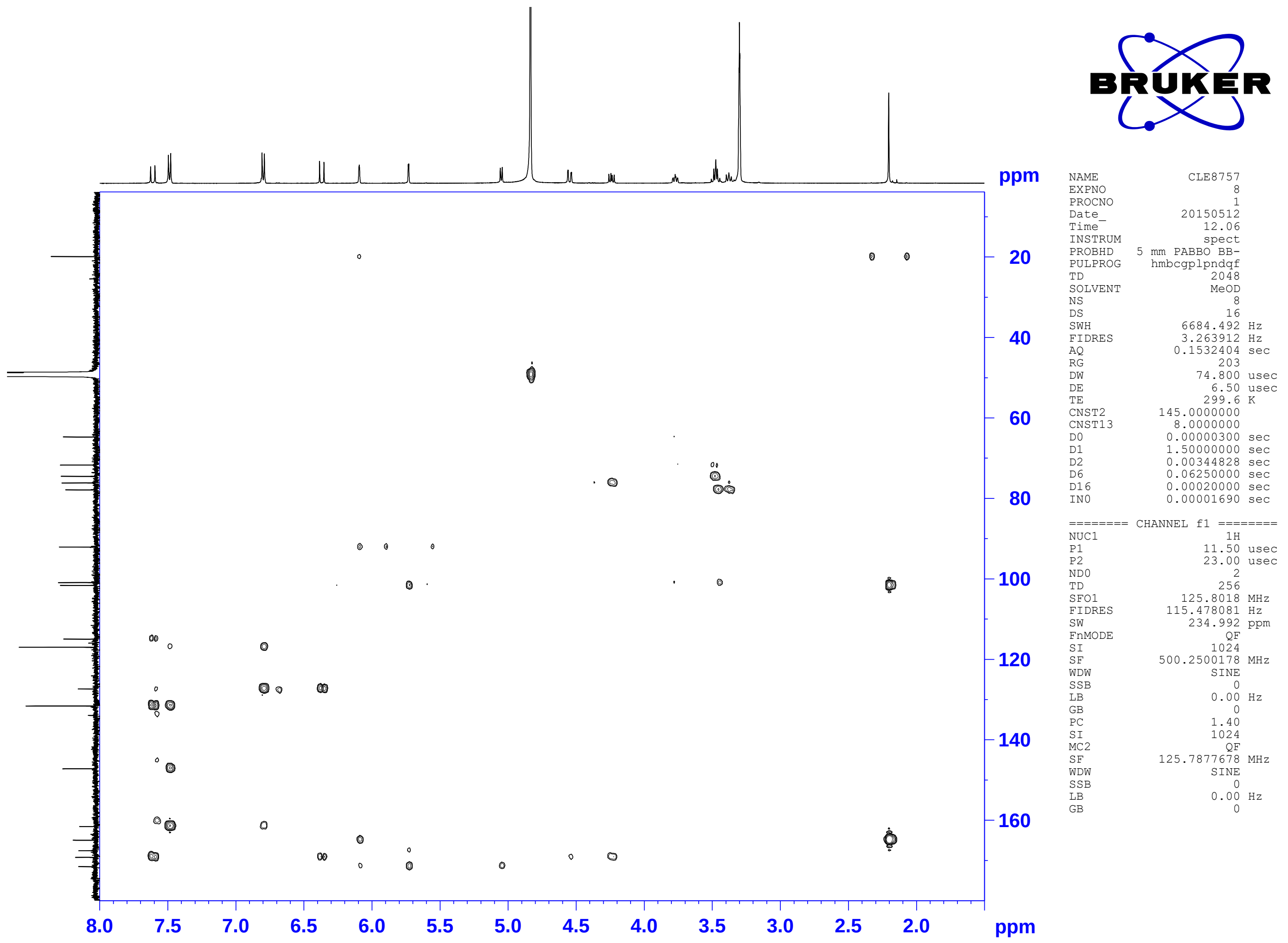

Figure 1-4S. HMBC spectrum of compound **1**.

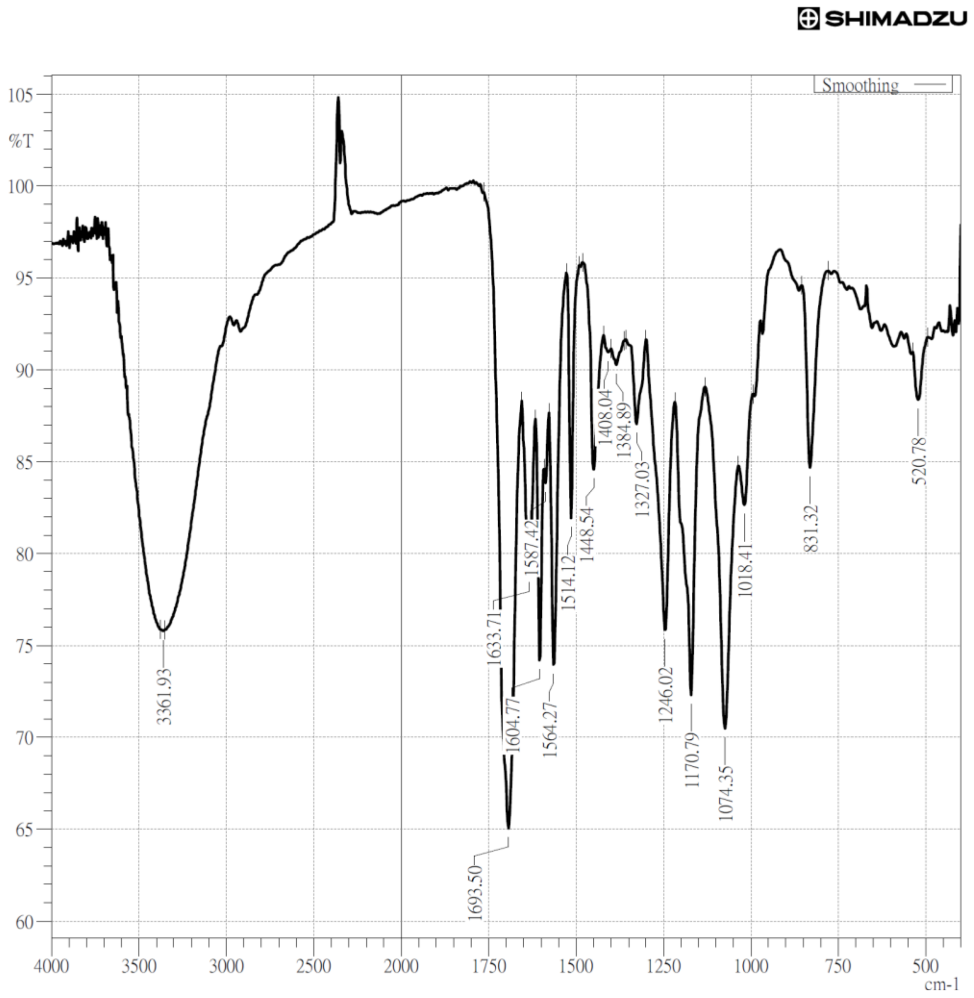

Figure 1-5S. IR spectrum of compound **1**.

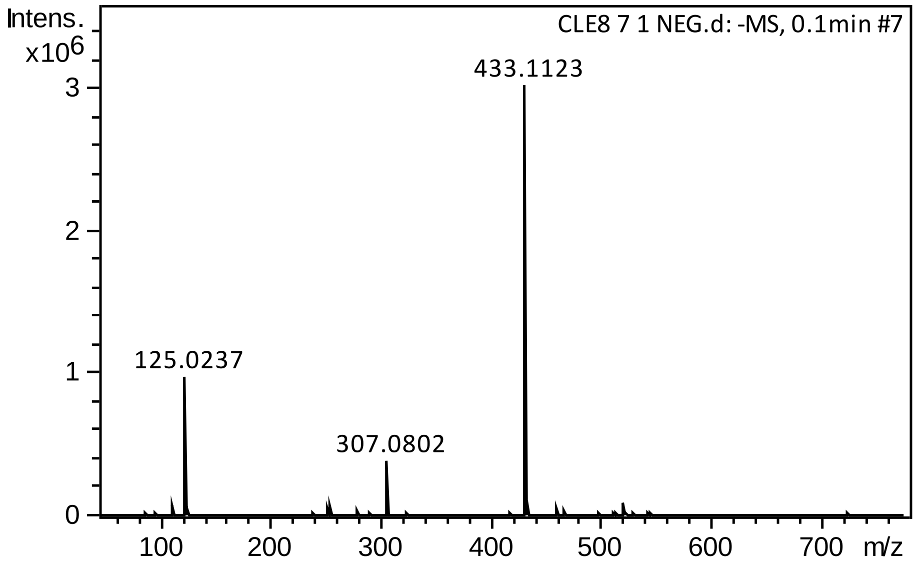

Figure 1-6S. HRESI MS spectrum of compound **1**.

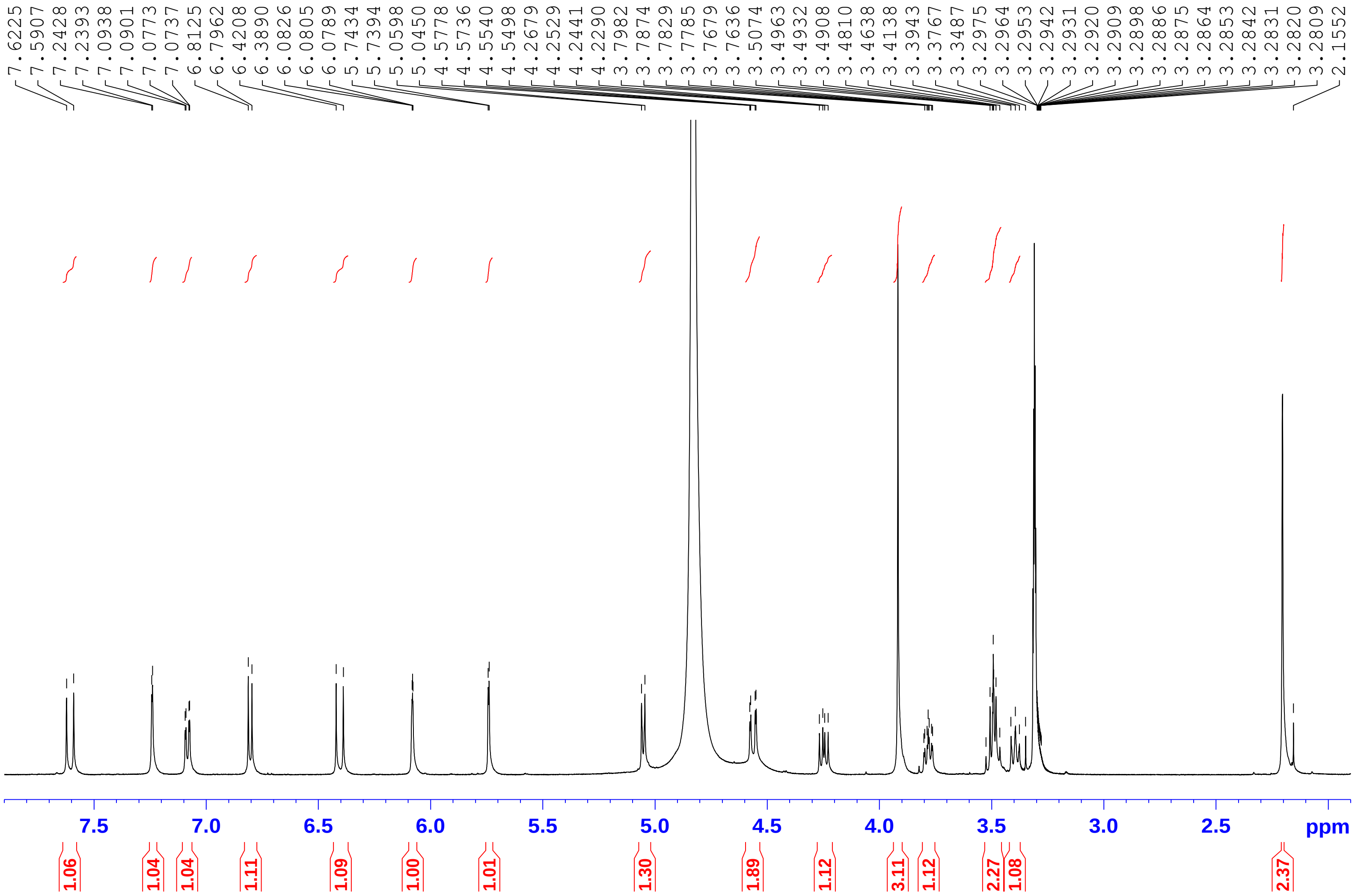

Figure 2-1S. ^{1}H NMR spectrum of compound **2** (CD_3OD, 500 MHz).

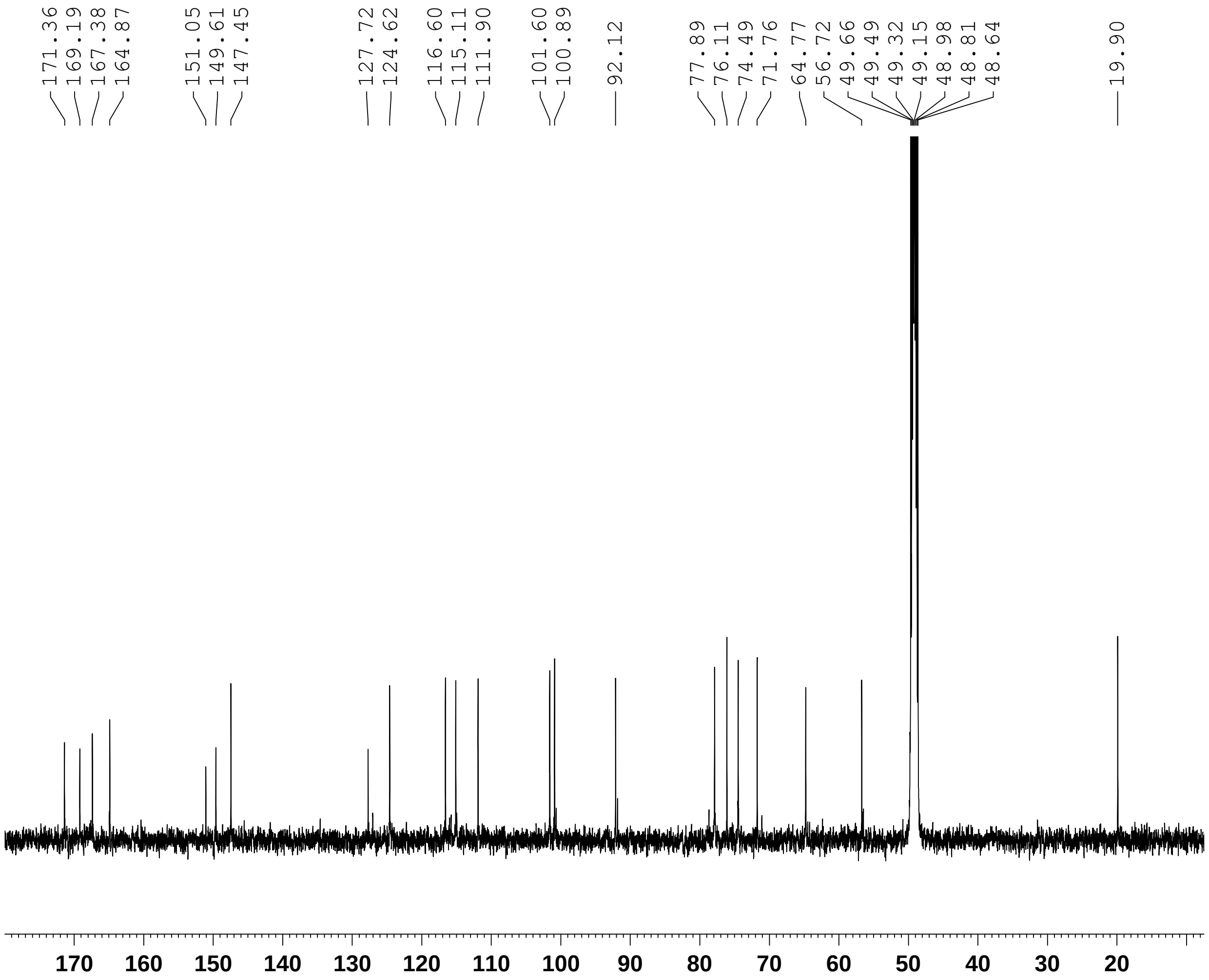

Figure 2-2S. ^{13}C NMR spectrum of compound **2** (CD_3OD, 125MHz).

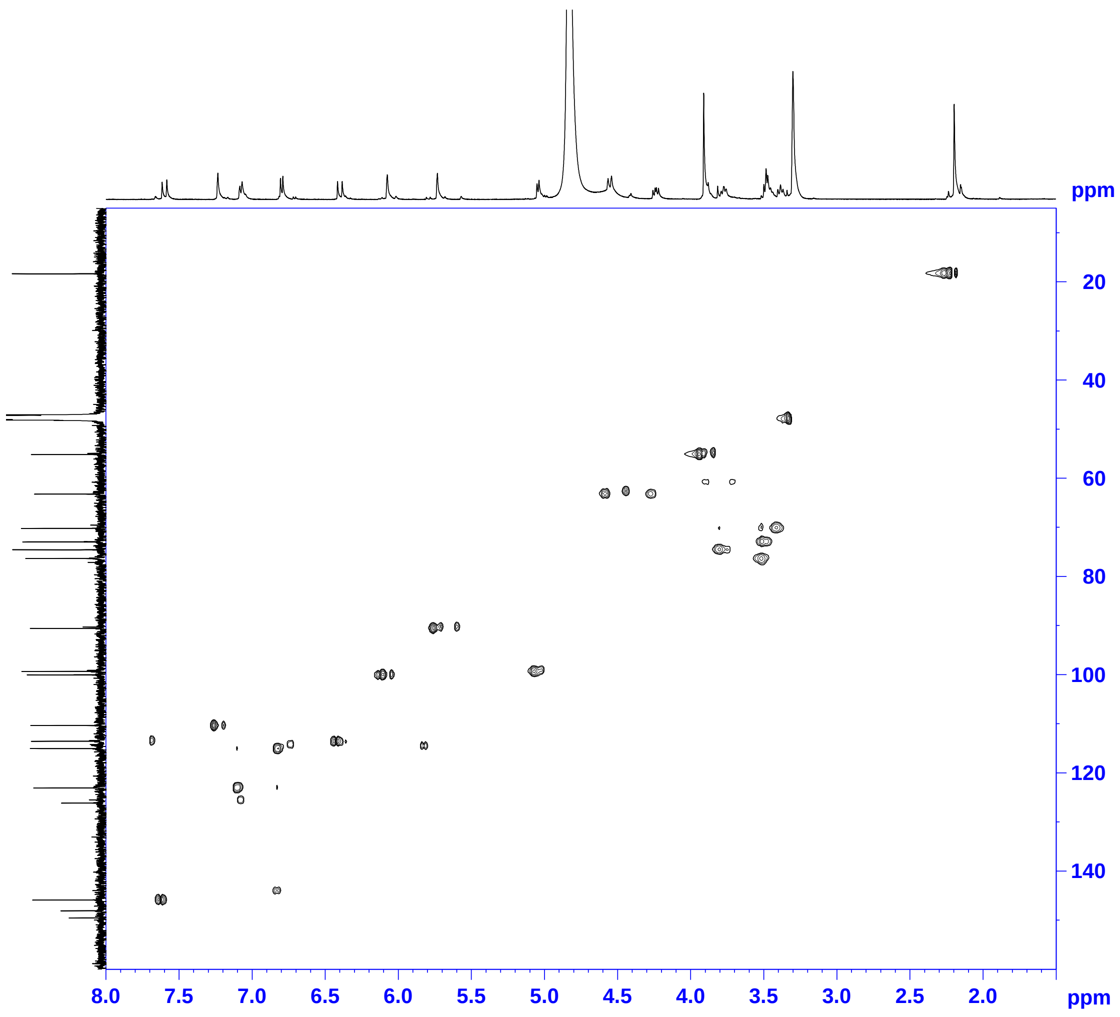

Figure 2-3S. HSQC spectrum of compound **2**.

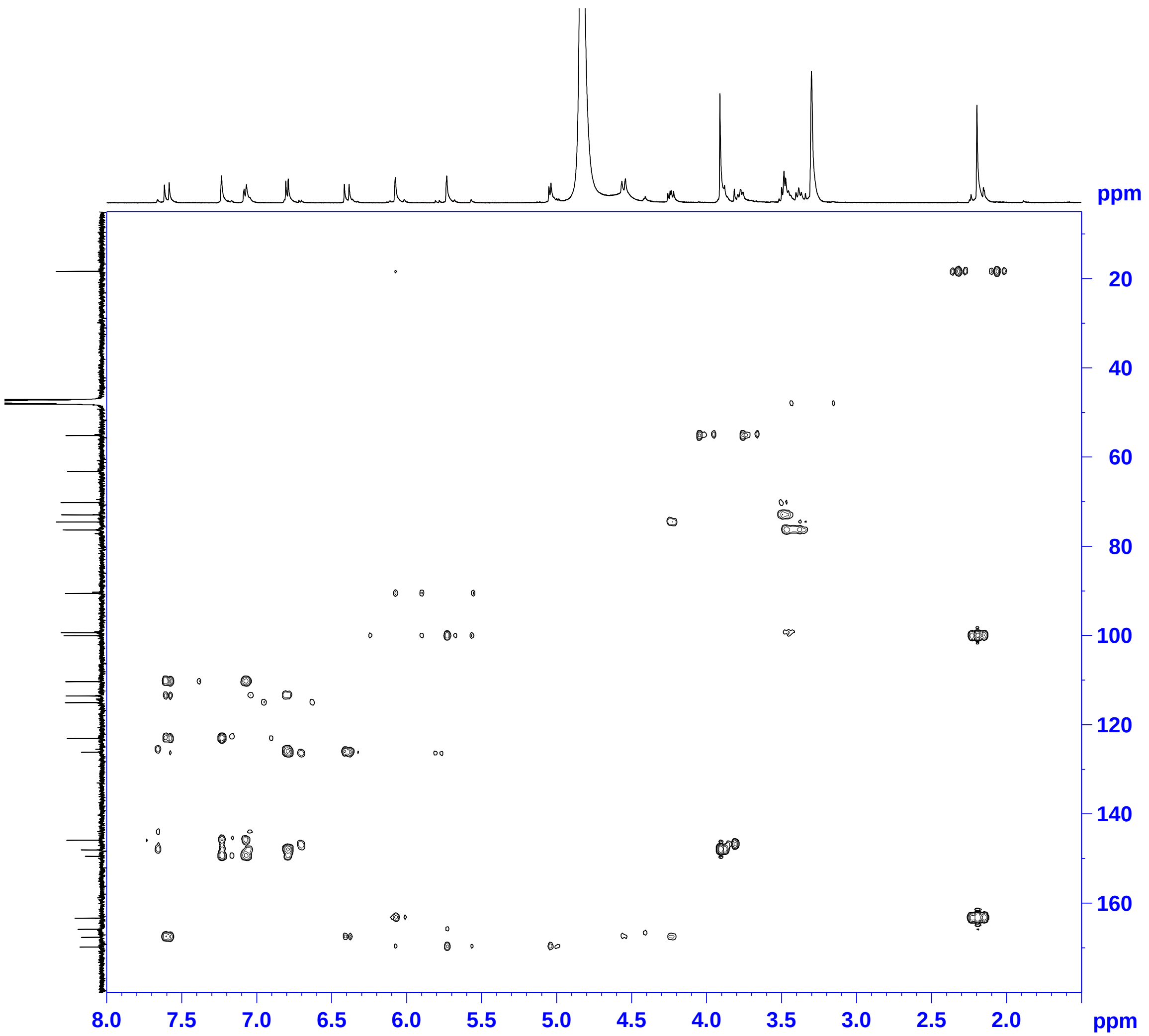

Figure 2-4S. HMBC spectrum of compound **2**.

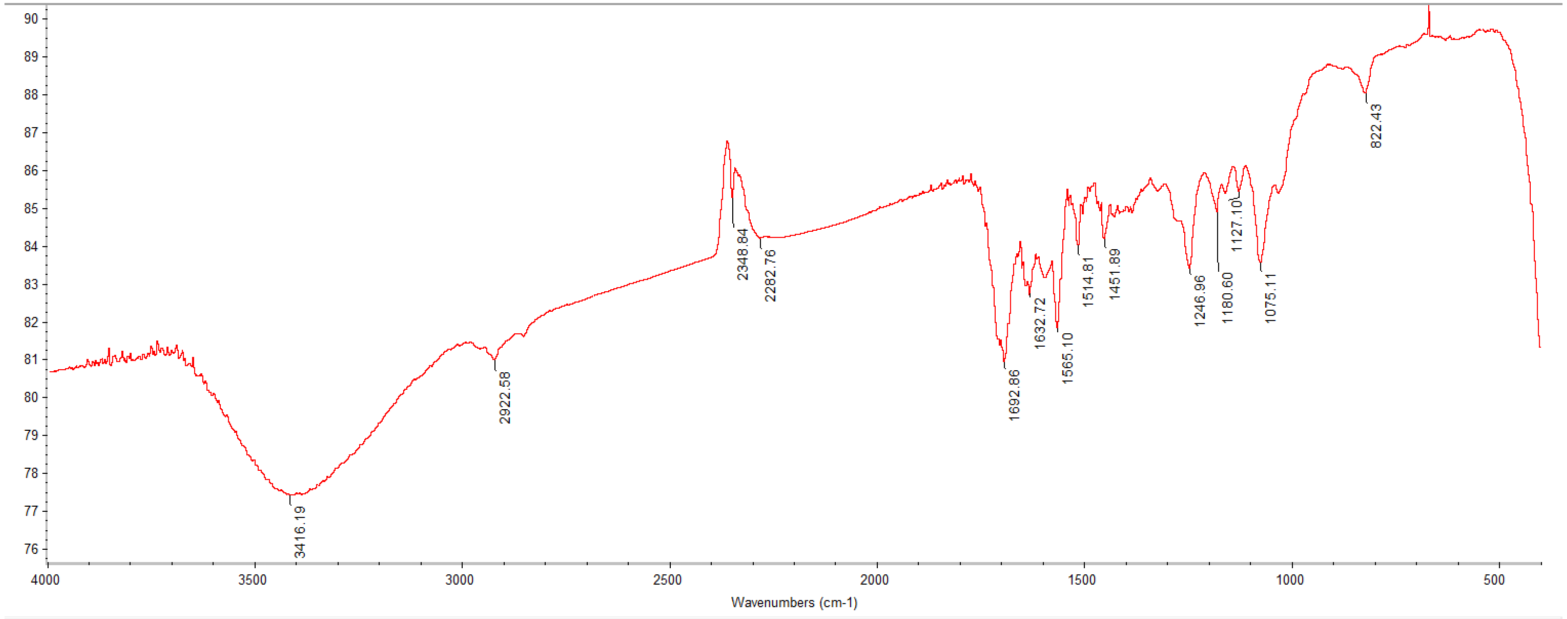

Figure 2-5S. IR spectrum of compound **2**.

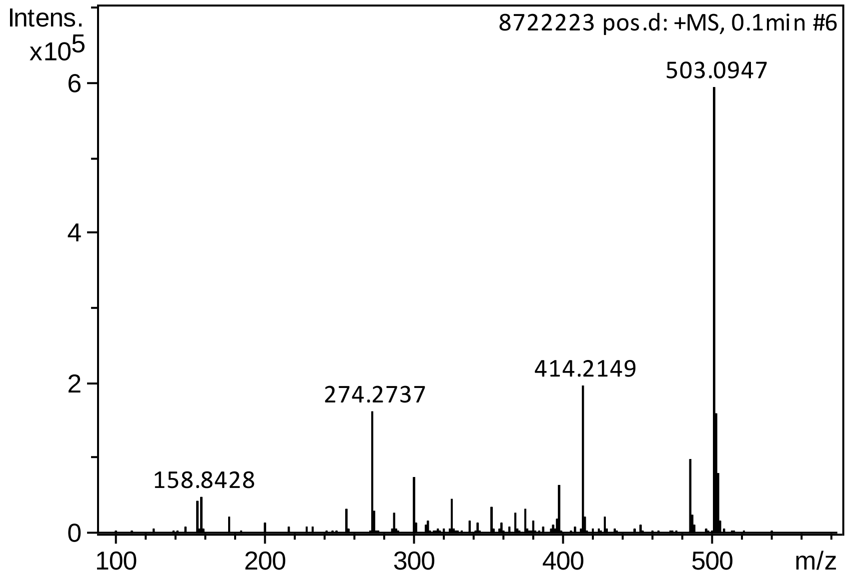

Figure 2-6S. HRESI MS spectrum of compound **2**.

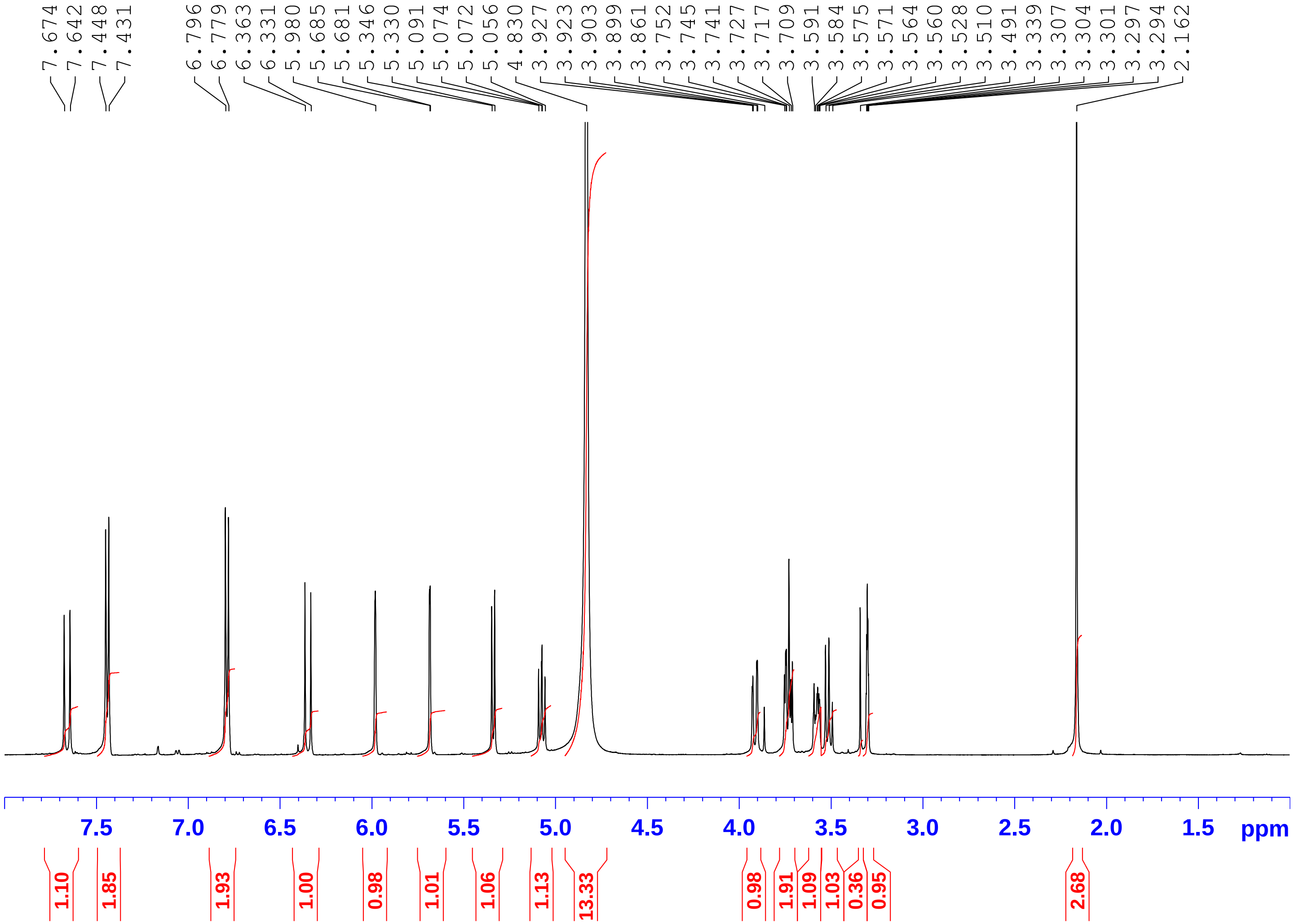

Figure 3-1S. ^{1}H NMR spectrum of compound **3** (CD_3OD, 500 MHz).

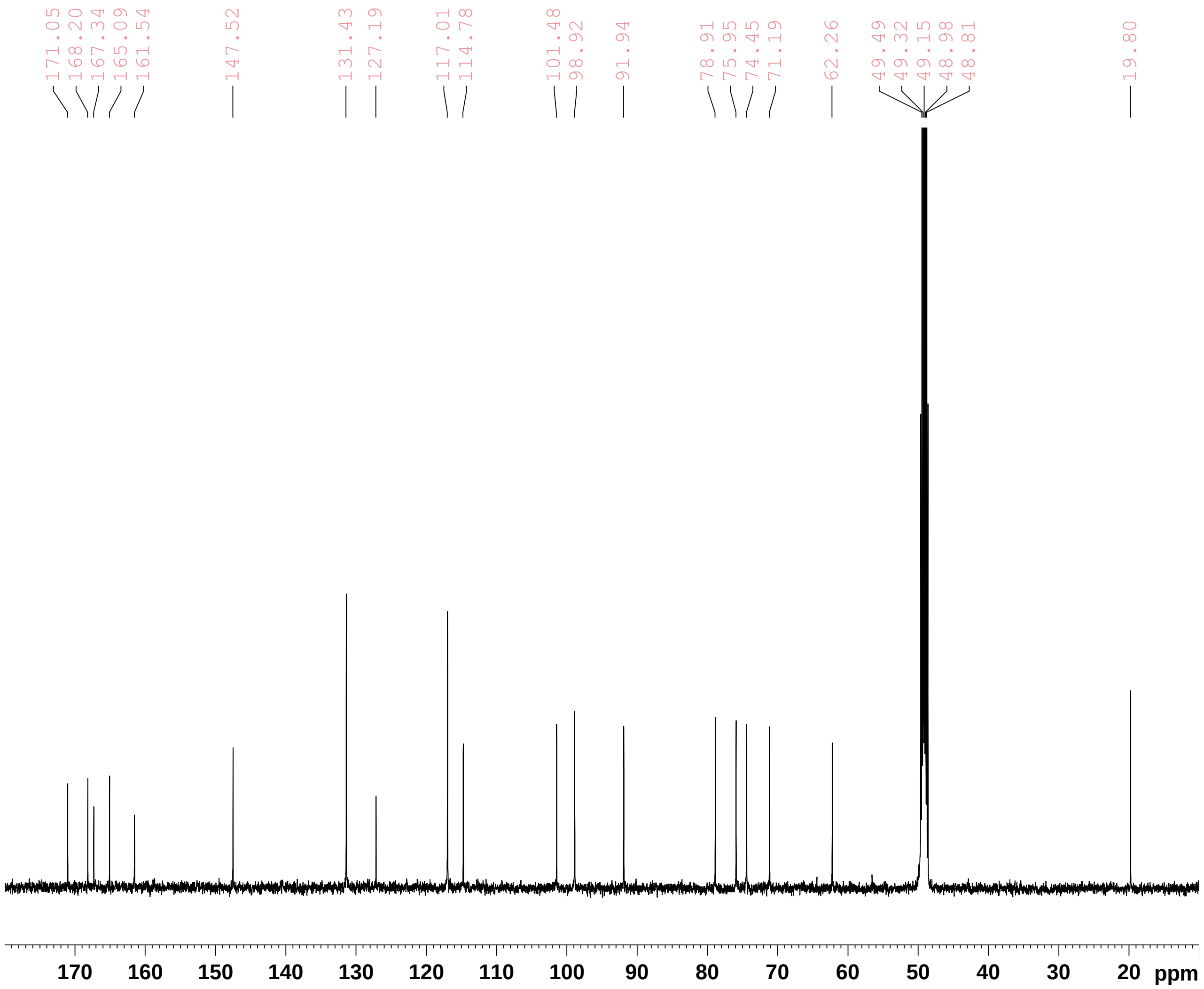

Figure 3-2S. ^{13}C NMR spectrum of compound **3** (CD_3OD, 125 MHz).

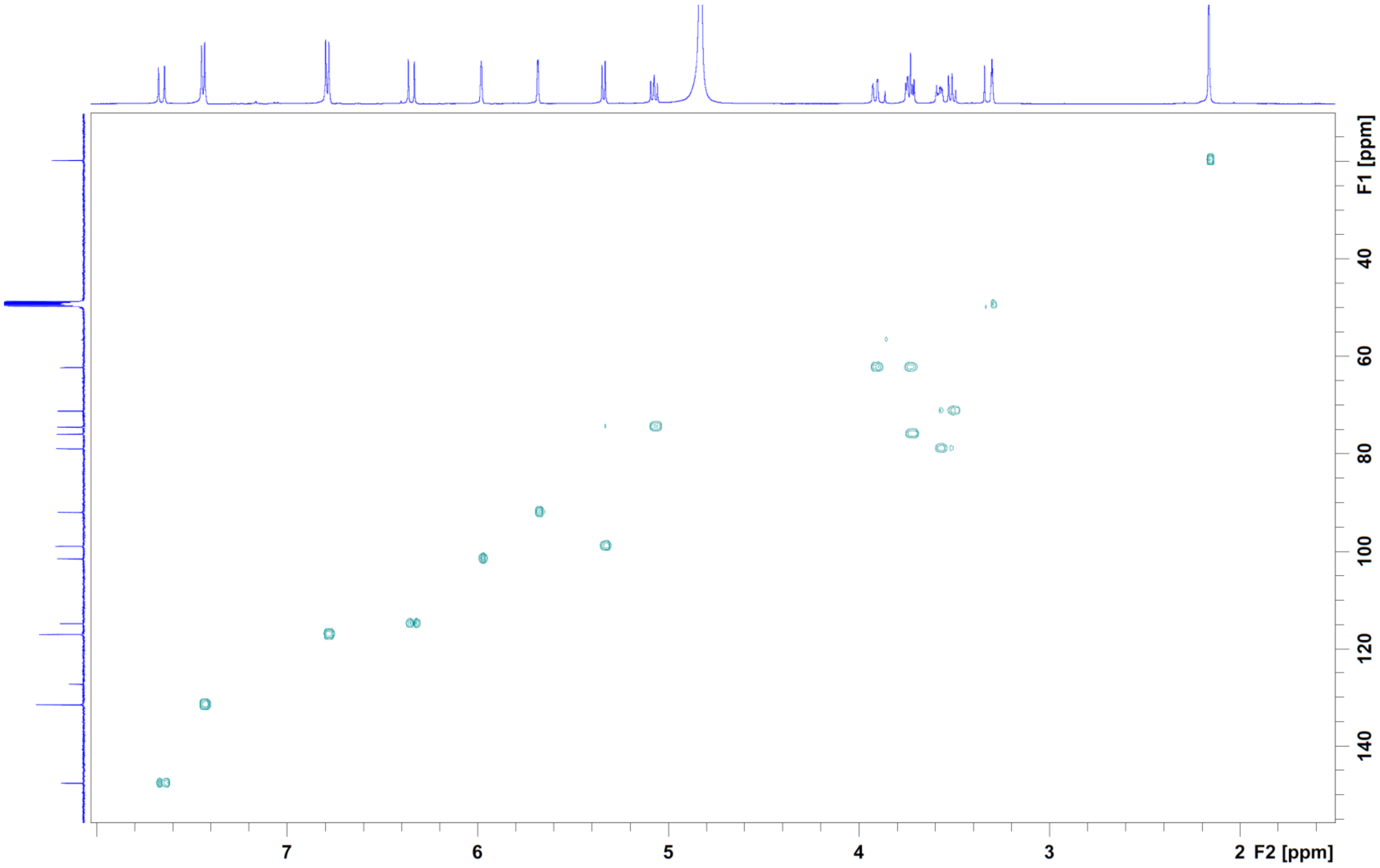

Figure 3-3S. HSQC spectrum of compound **3**.

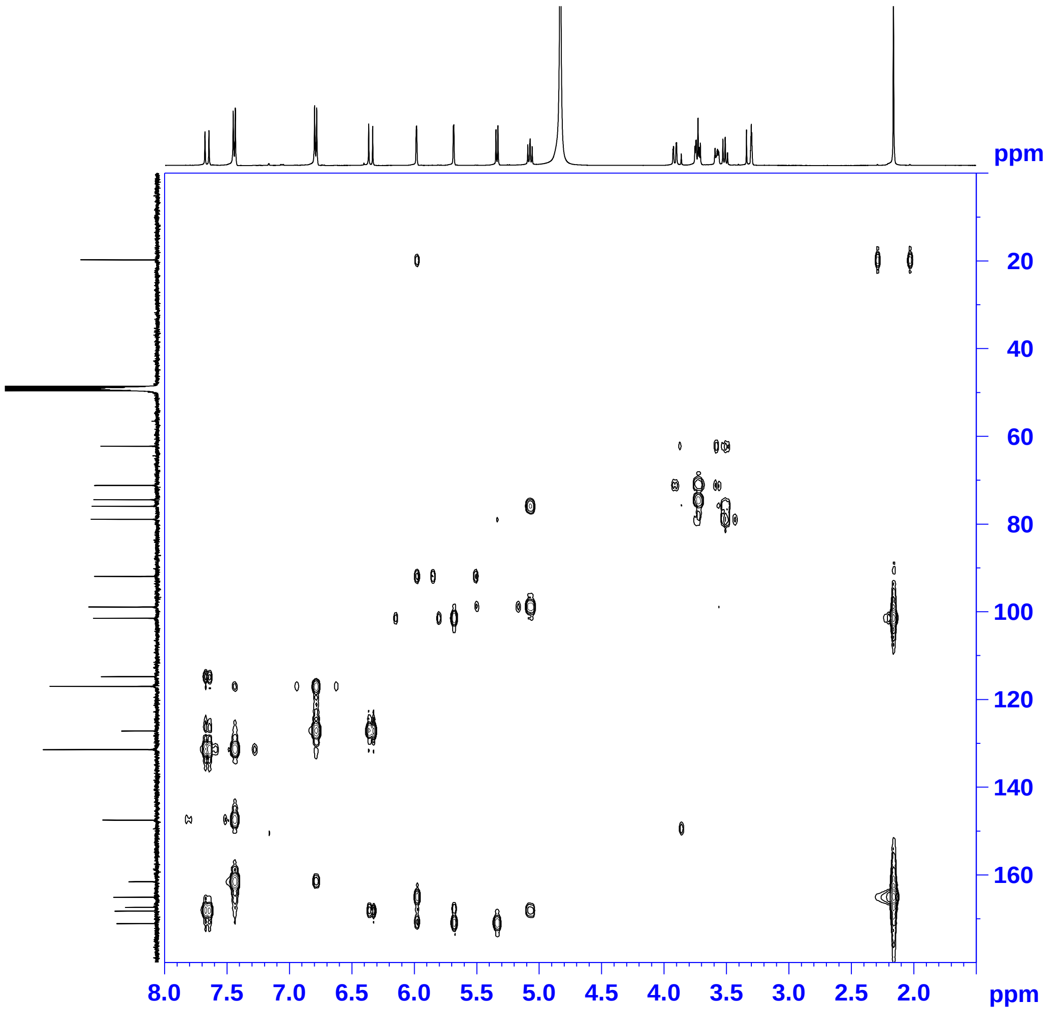

Figure 3-4S. HMBC spectrum of compound **3**.

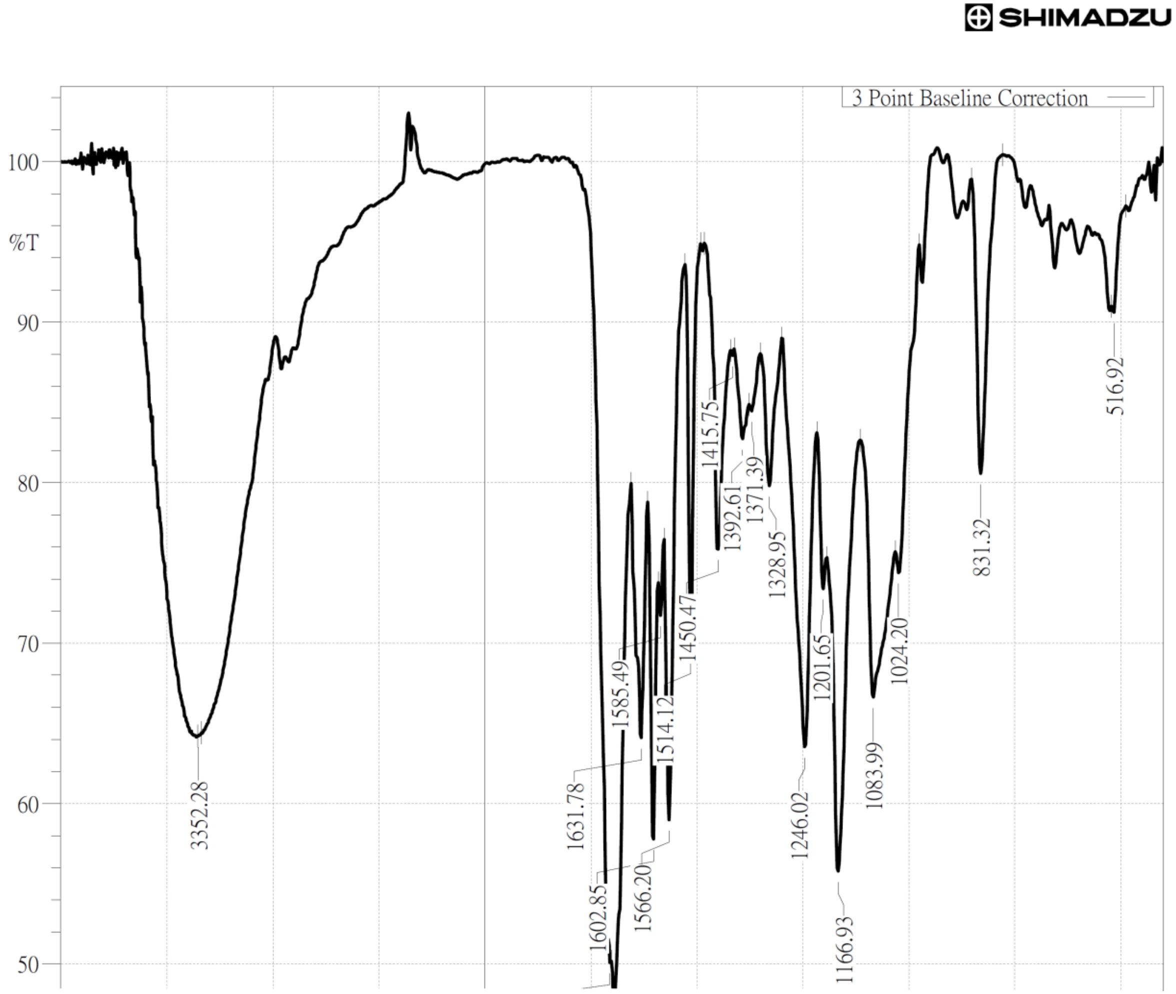

Figure 3-5S. IR spectrum of compound **3**.

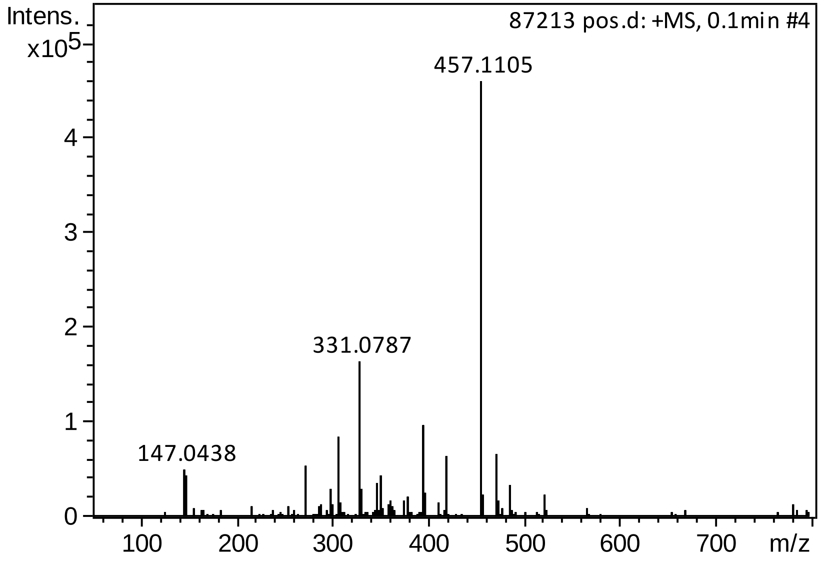

Figure 3-6S. HRESIMS spectrum of compound **3**.

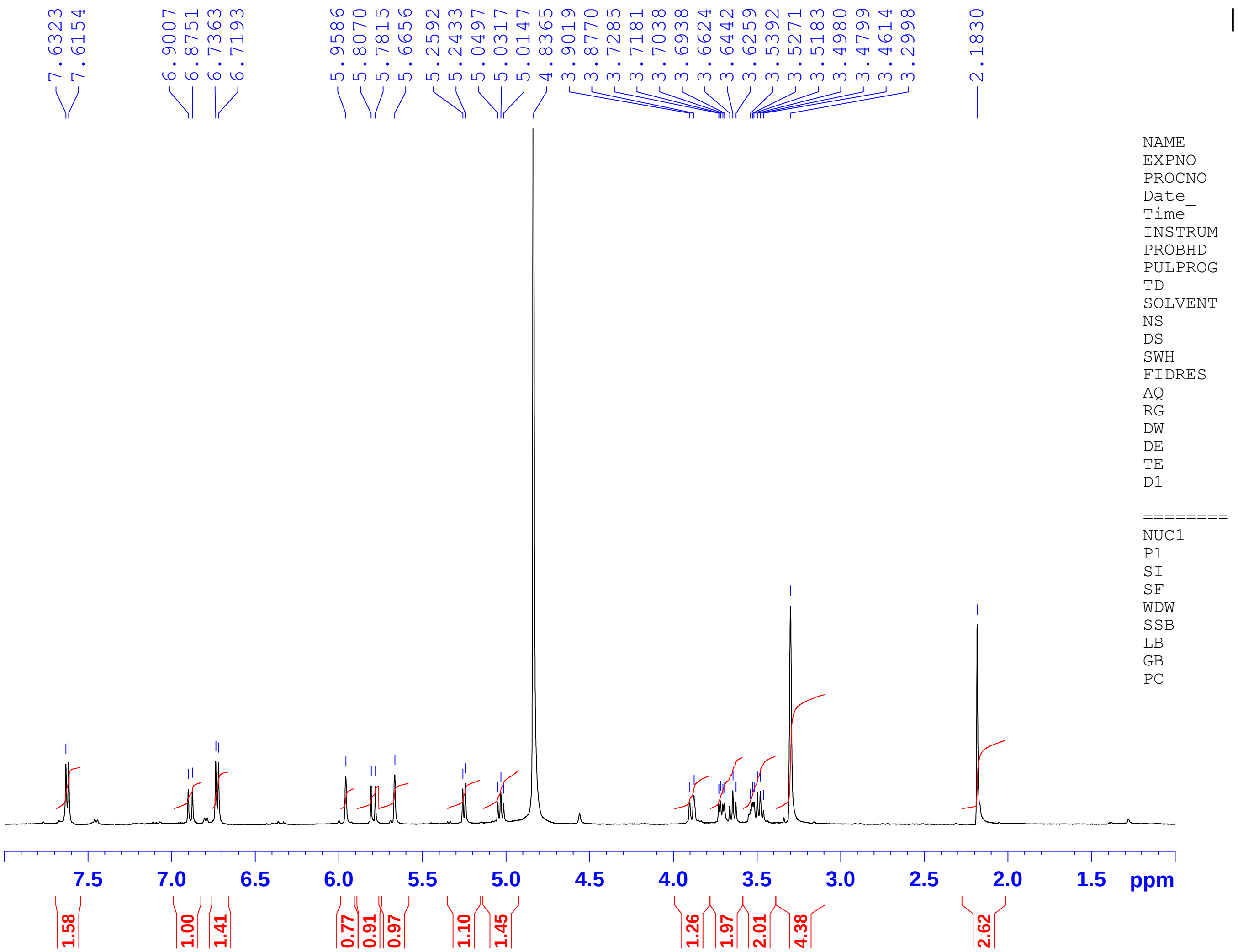

Figure 4-1S. ^{1}H NMR spectrum of compound **4** (CD_3OD, 500 MHz).

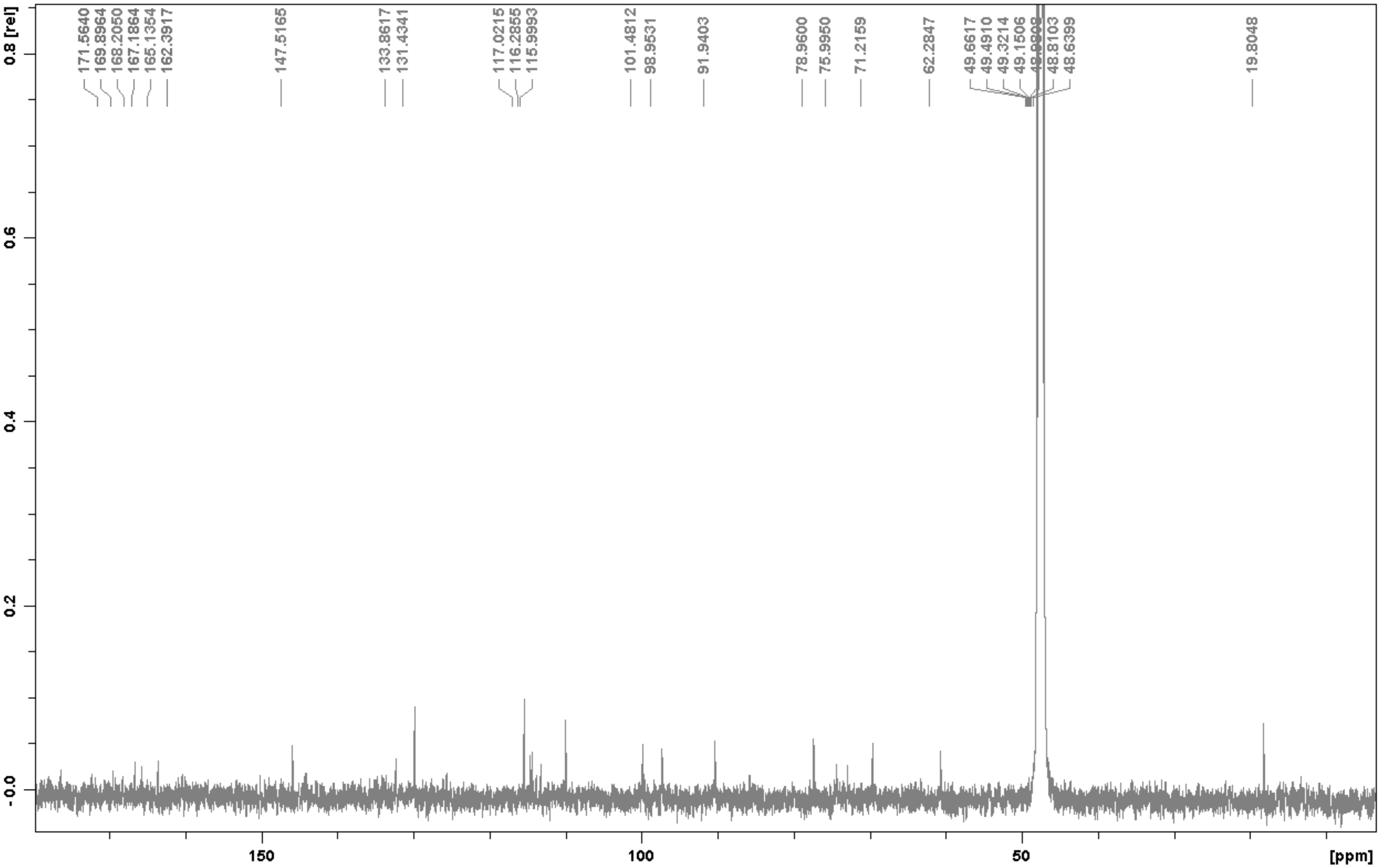

Figure 4-2S. ^{13}C NMR spectrum of compound **4** (CD_3OD, 125 MHz).

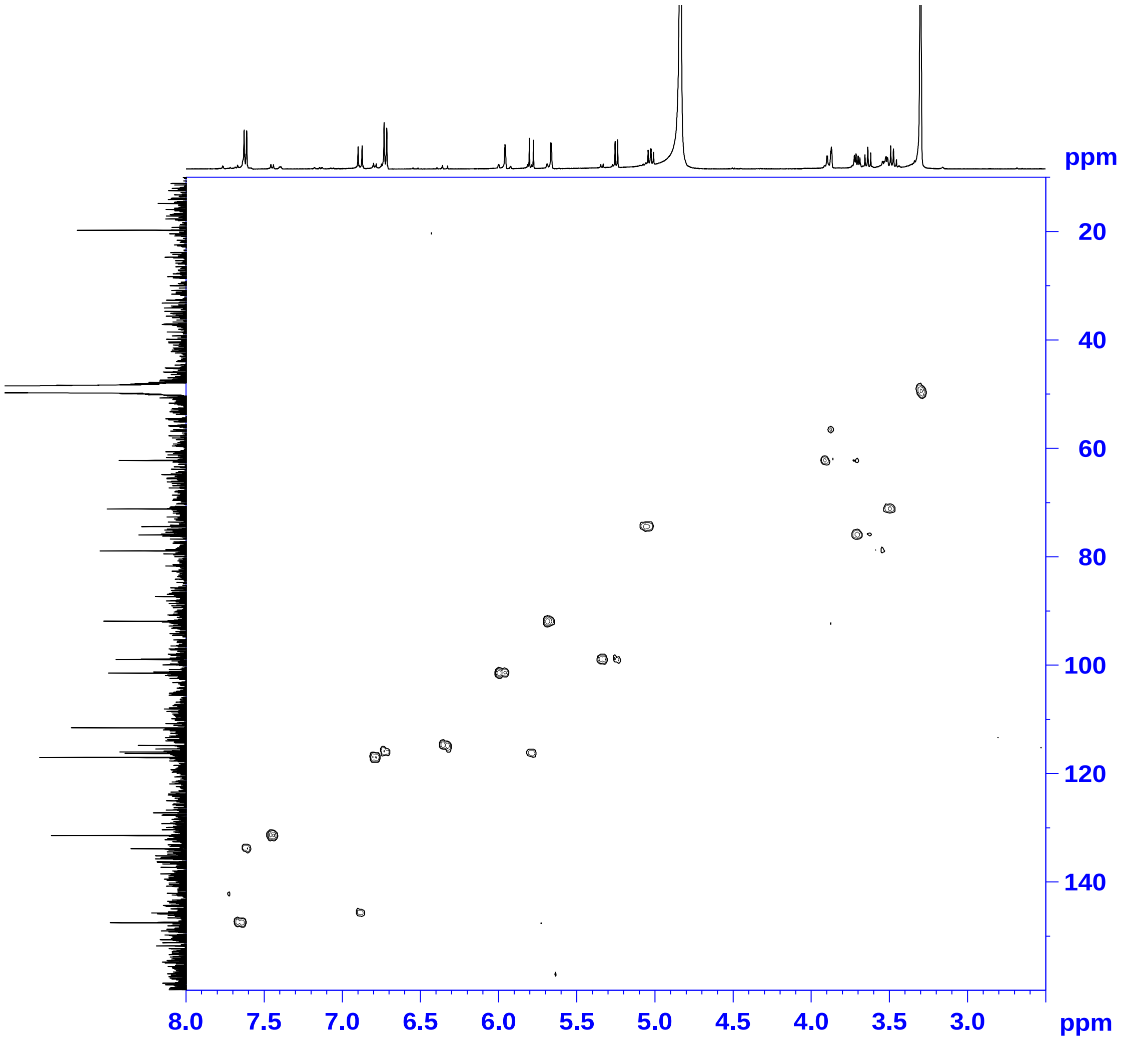

Figure 4-3S. HMQC spectrum of compound **4**.

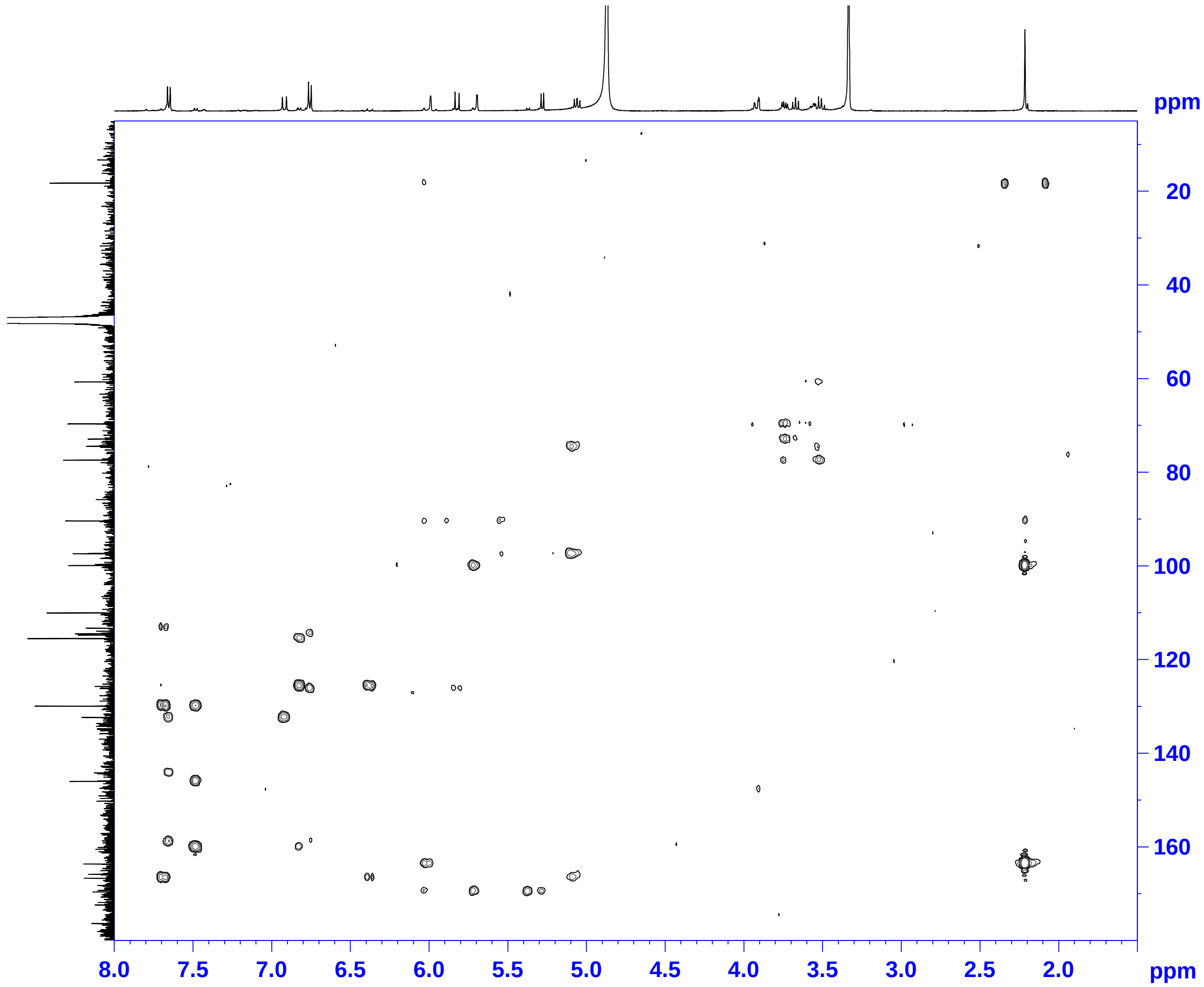

Figure 4-4S. HMBC spectrum of compound **4**.

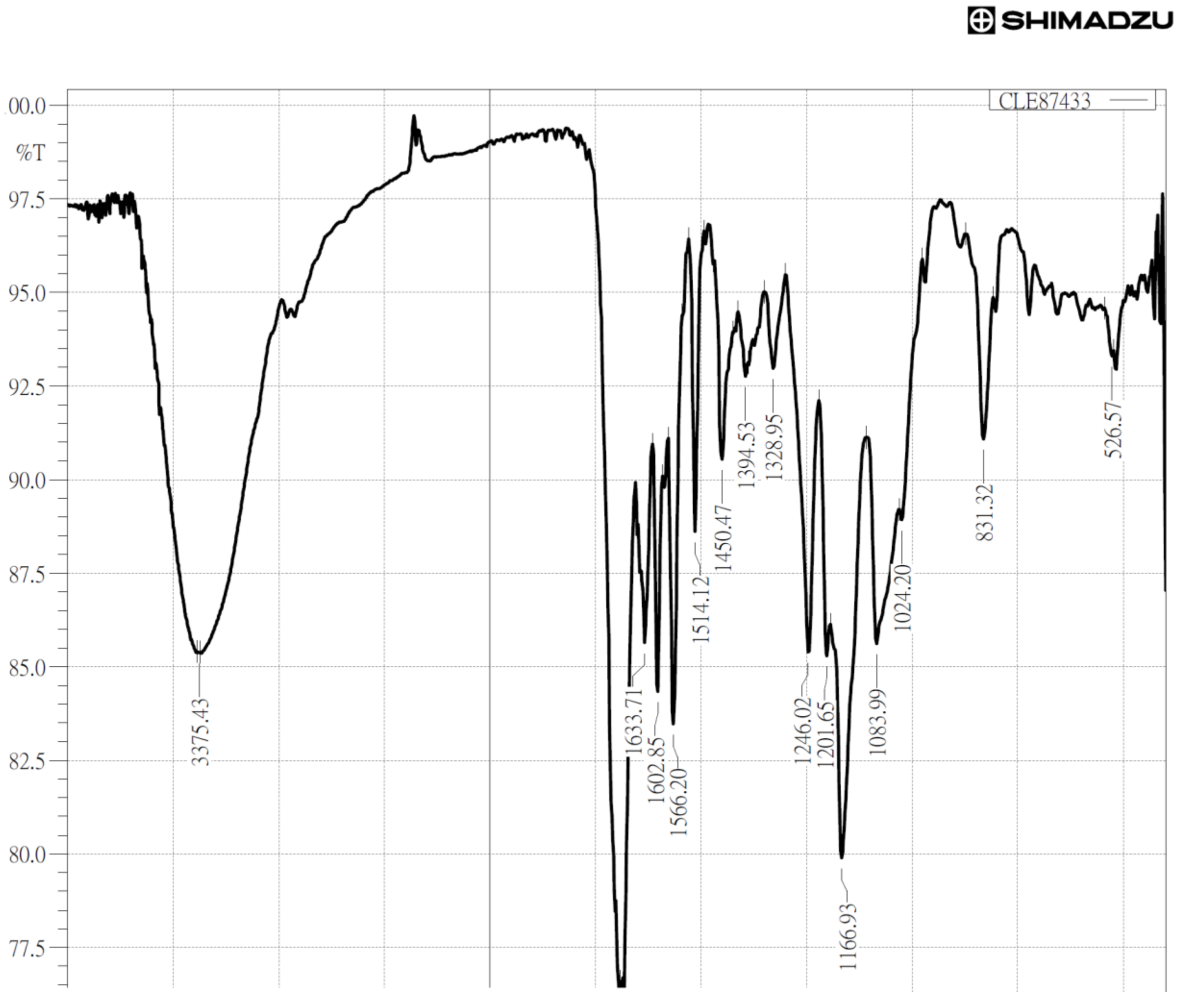

Figure 4-5S. RI spectrum of compound **4**

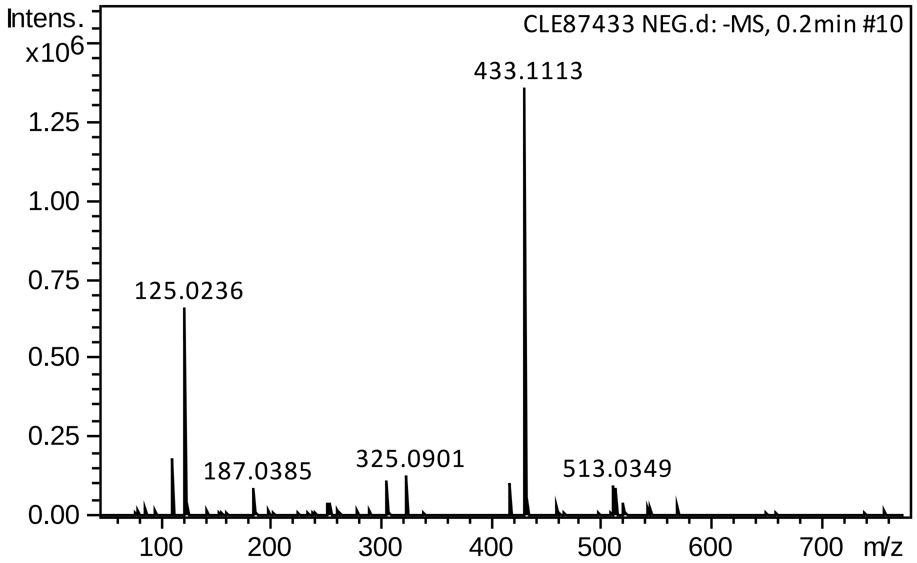

Figure 4-6S. HRESIMS spectrum of compound **4**
